# MicroRNAome Comparison between Intramuscular and Subcutaneous Vascular Stem Cell Adipogenesis

**DOI:** 10.1371/journal.pone.0045410

**Published:** 2012-09-20

**Authors:** Yunxue Guo, Delin Mo, Yue Zhang, Yun Zhang, Peiqing Cong, Shuqi Xiao, Zuyong He, Xiaohong Liu, Yaosheng Chen

**Affiliations:** State Key Laboratory of Biocontrol, School of Life Sciences, Sun Yat-sen University, Guangzhou, PR China; The Roslin Institute, University of Edinburgh, United Kingdom

## Abstract

**Background:**

As an important factor affecting meat quality, intramuscular fat (IMF) content is a topic of worldwide concern. Emerging evidences indicate that microRNAs play important roles in adipocyte differentiation. However, miRNAome has neither been studied during porcine intramuscular preadipocyte differentiation, nor compared with subcutaneous preadipocytes. The objectives of this study were to identify porcine miRNAs involved in adipogenesis in primary preadipocytes, and to determine whether intramuscular and subcutaneous adipocytes differ in the expression and regulation of miRNAs.

**Results:**

miRNAomes in primary intramuscular and subcutaneous adipocytes during differentiation were first sequenced using the Solexa deep sequencing method. The sequences and relative expression levels of 224 known (98.2% in miRbase 18.0) and 280 potential porcine miRNAs were identified. Fifty-four of them changed in similar pattern between intramuscular vascular stem cells (IVSC) and subcutaneous vascular stem cells (SVSC) differentiation, such as miR-210, miR-10b and miR-99a. Expression levels of 10 miRNAs were reversely up-or down-regulated between IVSC and SVSC differentiation, 19 were up-or down-regulated only during IVSC differentiation and 55 only during SVSC differentiation. Additionally, 30 miRNAs showed fat-depot specific expression pattern (24 in cells of intramuscular origin and 6 in cells of subcutaneous origin). These adipogenesis-related miRNAs mainly functioned by targeting similar pathways in adipogenesis, obesity and syndrome.

**Conclusion:**

Comparison of miRNAomes in IVSC and SVSC during differentiation revealed that many different miRNAs are involved in adipogenesis, and they regulate SVSC and IVSC differentiation through similar pathways. These miRNAs may serve as biomarkers or targets for enhancing IMF content, and uncovering their function in IMF development will be of great value in the near future.

## Introduction

The pig is one of the most important domesticated animals, and one of the major human nutritional sources of animal-based protein. To meet the increasing demand of consumers for pork quality, increasing the intramuscular fat (IMF) content while concomitantly decreasing backfat content is a major goal of animal scientists worldwide. IMF differs in a number of properties from the subcutaneous fat depots of animals. Intramuscular adipocytes mainly use glucose and acetate as energy sources, while subcutaneous adipocytes mainly use acetate [1], and triacylglycerol biosynthesis is less sensitive to starvation in intramuscular than subcutaneous adipose tissue [2]. Intramuscular adipocytes typically display rates of triglycerides biosynthesis that are 5 to 10% of those observed in subcutaneous adipose tissue [3], [4]. Though intramuscular adipocytes have lower expression levels of some proteins involved in anabolic and energy-yielding catabolic pathways than subcutaneous adipocytes, such as adipocyte lipid droplet binding protein (perilipin) and very long chain acyl-CoA dehydrogenase (VLCAD) [5], some proteins are more highly expressed in intramuscular adipocytes than subcutaneous adipocytes, such as bone morphogenetic proteins (BMP) 4 and 7 [6]. Currently, little is known about how these mRNAs and proteins are regulated in intramuscular and subcutaneous adipocytes.

MicroRNAs (miRNAs), 18–26nt non-coding RNAs, are essential regulators of diverse biological processes including lipid metabolism and preadipocyte differentiation, and they function by targeting ∼30% of mRNAs or proteins [7]–[9]. Understanding the mechanisms by which miRNAs regulate adipogenesis is likely to provide new perspectives on adipose tissue development [10]. The first miRNA reported to be involved in adipose cell biology was miR-143 [11], which positively regulates preadipocyte differentiation. Soon afterwards, some other miRNAs involved in adipogenesis *in vitro* and *in vivo* were found, such as miR-210 [12], the miR-17-92 cluster [13], miR193b-365 [8]. The miRNAs were found to involve in adipogenesis mainly using microarray or solexa deep sequencing method [8], [12]. However, unlike neuronal and muscle tissues, much less information is known about the role of miRNA in adipose tissue, especially those from different fat-depots of same individual.

How miRNAs regulate adipogenesis is of considerable interest for animal scientists. Some miRNAs have been identified as adipose tissue-specific or significantly changed during adipogenesis in most major livestock species, such as bovine [14] and pig [10], but they focused on adipose tissues consisting of a heterogeneous mixture of cell types. Only 228 swine miRNAs sequences have been submitted to miRBase (18.0), far fewer than those for bovine, chicken, and other livestock species, the sequences were mainly detected in skeletal muscles. To the best of our knowledge, no work has yet been conducted to determine miRNA changes during primary swine intramuscular vascular stem cells (IVSC) and subcutaneous vascular stem cells (SVSC) differentiation, though differences at mRNA and protein level for the two types of adipogenesis is partially known.

MiRNA expression patterns in undifferentiated and differentiated porcine intramuscular and subcutaneous vascular stem cells were obtained by Solexa sequencing. The objectives of this research were to identify porcine miRNAs involved in adipogenesis in primary preadipocytes, and to determine whether intramuscular and subcutaneous adipocytes differ in the expression and regulation of miRNAs. Dynamic changes of miRNAs and mRNAs were found during IVSC and SVSC differentiation, miRNAs involved in adipogenesis in primary intramuscular and subcutaneous adipocytes differed in expression were observed. Making full use of the results may provide new strategies for increasing IMF content of pigs.

## Results

### Differentiation of SVSC and IVSC

**Figure 1:**
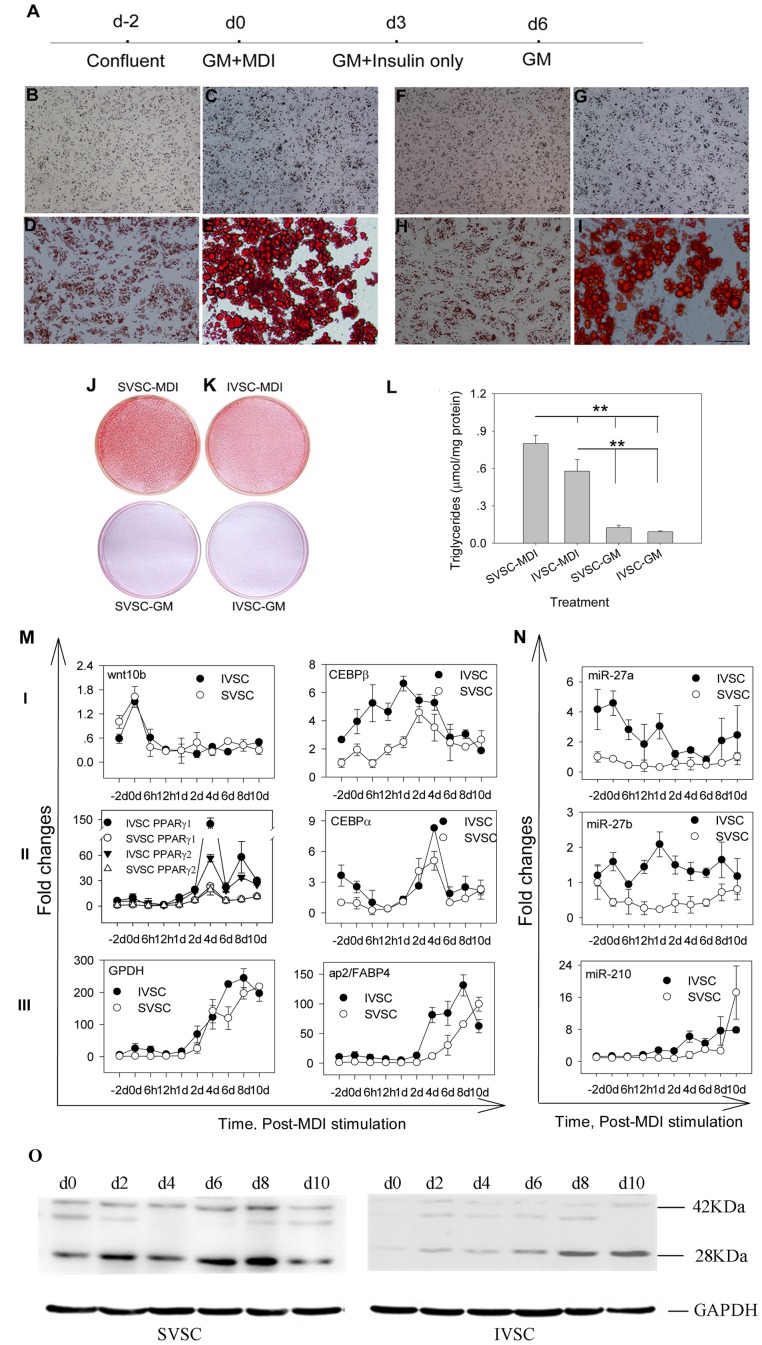
Adipogenic potency of SVSC and IVSC. The overall experimental scheme is illustrated in A. SVSC and IVSC differentiation was induced at d0, the cells (B-E, J represent SVSC; F-I, K represent IVSC; B-D and F-H, 100×; E and I 400×) were fixed and stained with oil red O at d3 (B; F,), d6 (C; G,) and d9 (D, E, J; H, I, K). Triglycerides contents in SVSC and IVSC in growth medium (GM) and differentiation medium (MDI) at d10 were also determined. Replicates were the same as L (L). Adipogenic-related mRNAs and miRNAs were quantified by qPCR at different times after MDI stimulation (M and N, respectively). Date were first normalized to *GAPDH* mRNA for each mRNA and miRNA, then fold changes relative to their respective levels in IVSC preadipocyte (day -2) were calculated, replicates were the same as L. C/EBPα protein levels during SVSC and IVSC differentiation were determined by western blotting, GAPDH protein was used as endogenous control (O).

To determine differentiation potency of primary IVSC and SVSC into adipocytes, the cells were induced by MDI (0.5 mM 3-isobutyl-1-methylxanthine, 0.25 µM dexamethasone, and 5 mg/l insulin) or left untreated according to the schemes in Fig.1A and methods in Text S1. Lipid droplets were visible under microscope after 3 days’ induction (Fig.1B and F), and more lipids were accumulated later (Fig.1C and G). By day nine, more and larger visible lipid droplets were accumulated, indicating the primary cells were differentiated into mature adipocytes (Fig.1D, E, H, I and J, K). IVSC could accumulate less triglycerides than SVSC (Fig. 1L). Similar dynamic curves of adipogenic mRNAs and miRNAs were observed during IVSC and SVSC differentiation (Fig. 1M and N); the mRNAs were Pearson correlated between IVSC and SVSC (*p*<0.01) except for *C/EBPβ*. The early marker *wnt10b* showed significantly higher expression at d0 than other time points (*p*<0.05). *C/EBPβ* was more highly expressed in IVSC than SVSC at most time points (Fig. 1M). The essential adipogenic - *PPARγ1*, *PPARγ2* and *C/EBPα* - were first up-regulated and then down-regulated, and all of them were most highly expressed on day 4 (Fig. 1M).

The adipocyte-specific *GPDH* and *ap2* were up-regulated during the whole differentiation process, especially after day 2 (Fig. 1M). MiR-27a and miR-27b were more highly expressed in IVSC than SVSC, and expressions of both miRNAs were lower in mature adipocytes than preadipocytes (Fig. 1N). MiR-210 was up-regulated throughout the differentiation of both IVSC and SVSC (Fig. 1N). The C/EBPα protein level increased up to day 8 in SVSC and day 10 in IVSC after MDI stimulation (Fig. 1O). Judging from the phenotype and molecular changes above, the primary IVSC and SVSC could differentiate into mature adipocytes efficiently.

### Small RNA Sequencing

To identify small RNA changes during swine IVSC and SVSC differentiation, and to compare whether the change is origin-specific, a Solexa deep sequencing experiment was carried out during differentiation of VSC isolated from dorsal longissimusdorsi and subcutaneous fat tissue. High quality unique reads were obtained in IVSC (27,468,545), IVSC-MDI (28,195,415), SVSC (33,496,442) and SVSC-MDI (13,019,273) small RNA libraries. The sequences detected in IVSC, IVSC-MDI, SVSC and SVSC-MDI could mapped to 97.4, 96.9, 94.7 and 94.3% of the miRNAs in miRbase (18.0) were respectively, meaning that Solexa sequencing was deep enough to discover nearly all of the porcine miRNAs in miRbase. MiRNAs accounted for 84.4, 83.2, 83.0 and 80.5% of the annotated reads, which means they were predominant annotated sequences. Of the small RNA sequences identified in IVSC (167, 40), IVSC-MDI (178, 40), SVSC (207, 63) and SVSC-MDI (124, 17), 72.3, 72.7, 75.4 and 74.1% were unannotated, meaning that unidentified small RNAs including miRNAs may exist in our libraries.

To examine the small RNA cDNA libraries were constructed successfully and the coverage of the deep-sequencing results, the data obtained from a library were comprehensively analyzed in terms of the numbers of unique small RNAs and the percentages of annotated small RNAs (Fig. S1). Although the numbers of unique small RNAs and the percentages of annotated small RNAs were far from exhausted after 28,200,000 total reads, the percentages of annotated read and miRbase found all began to plateau when the total reads reached 9,500,000. Hence, the data sets obtained from deep-sequencing of preadipocytes and adipocytes were all extensive enough to capture the diversity of miRNA abundance and evaluate relative steady state levels in the differentiation process examined. Similar saturation plots results were created from other libraries (data not shown).

### Small RNA Length Distribution

**Figure 2:**
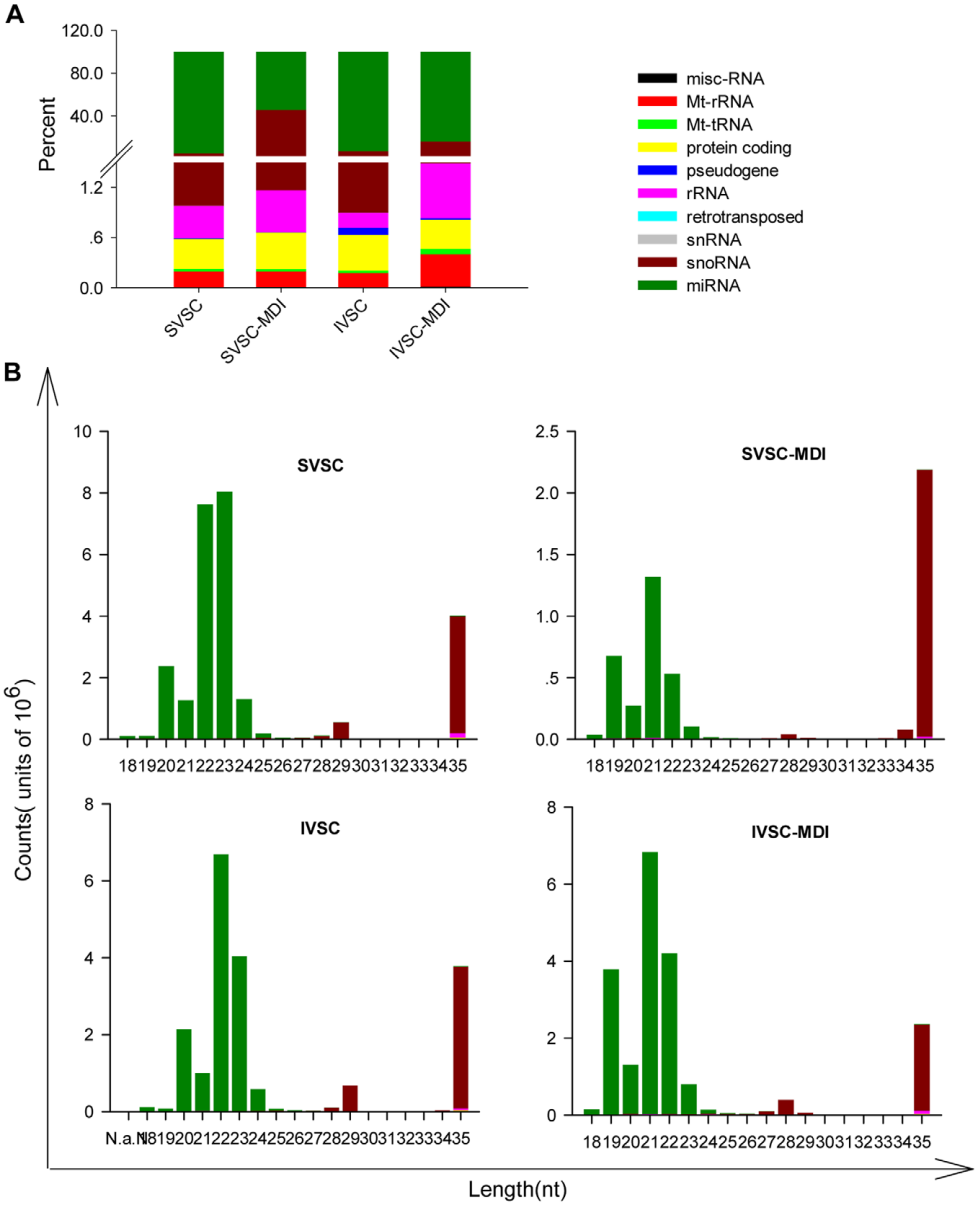
Composition of different classes of small RNAs in the four libraries. Miscellaneous RNAs (misc-RNA), microRNAs (miRNA), mitochondrial ribosomal RNAs (MT-rRNA), mitochondrial transfer RNAs (MT-tRNA), protein coding RNAs, pseudogenes, small nucleolar RNAs (snoRNA), ribosomal RNAs (rRNA), retrotransposed and small nuclear RNAs (snRNAs) were categorized in SVSC, SVSC-MDI, IVSC, IVSC-MDI libraries. A: percentages of the different classes of small RNAs in each library. B: small RNA length distribution in the above four libraries.

To study the complexity of, and changes in, all the small RNA classes during IVSC and SVSC differentiation, the abundances and length distributions of 18–35nt RNAs in the four libraries were assessed. IVSC, IVSC-MDI, SVSC and SVSC-MDI had 15,658,532, 20,241,400, 21,840,487 and 5,319,126 total reads were mapped to pig genome respectively. Sequences of 18–24nt and 35nt accounted for most reads (Fig. 2A, Figure. S2), making miRNA be the largest small RNA component in all the sequenced samples, which were 14,584,960, 17,071,026, 20,821,871 and 2,345,029 reads respectively. As the second largest component, snoRNA were 933152, 2888966, 804177 and 2912105 reads respectively (Fig. 2A and B). In the shorter lengths (18–24nt in SVSC and IVSC, 18–26nt in SVSC-MDI and IVSC-MDI), miRNA was the predominant small RNA class, while in the longer length (27–35nt), snoRNA was the most abundant (Fig. 2B). Not only miRNAs, but also other small RNA classes changed differently during SVSC and IVSC differentiation (except protein coding and snRNA) (Fig. 2A). The predominant miRNA and snoRNA changed more during SVSC than IVSC differentiation, while rRNA changed more during IVSC differentiation (Fig. 2A).

### Single Nucleotide Variation in Small RNAs

**Figure 3:**
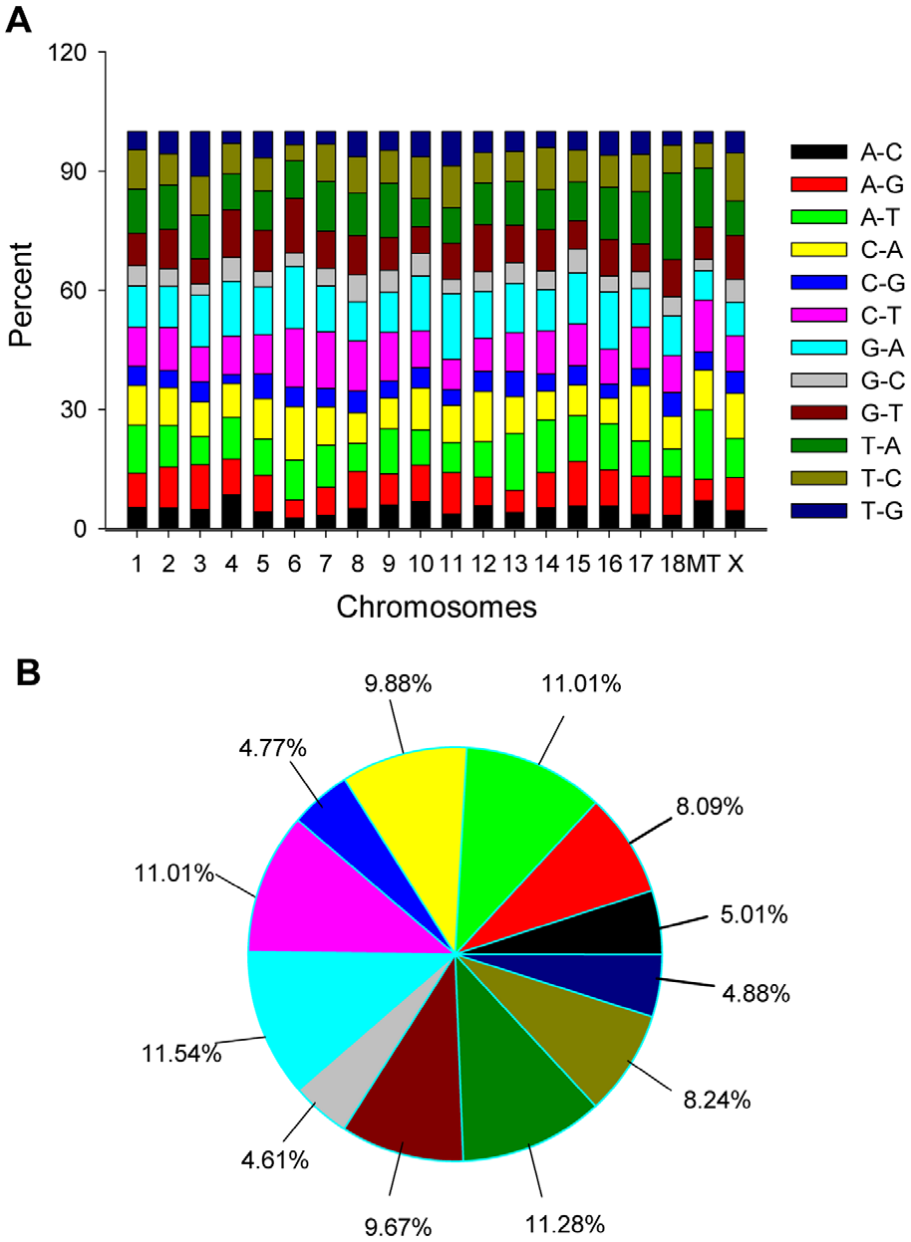
Single nucleotide variation in small RNA. There are 12 single nucleotide substitution types, and the former letter in each type represents the reference nucleotide in the porcine genome, with the last letter indicating the nucleotide identified in the sequencing results. The percentage of each type in all the chromosomes and mitochondria (MT) were calculated (A). Integrated results of each nucleotide substitution type in the porcine genome are shown in B; the numbers indicate percentage substitutions.

Considering that only <20% of small RNAs were annotated in the present study (Fig. S1), all sequences were mapped to pig genome to determine the percentages of each type of substitution in all small RNAs, and correlation analysis of their percentages in each chromosome and mitochondria (MT) were performed using SPSS 16.0. Similar results were obtained in the four libraries. The percentage of each type of nucleotide substitution was similar in any chromosome and MT, and most of them were Pearson correlated (*p*<0.0001) (Fig. 3A). In the whole genome, G-A showed the largest percentage (11.54%), followed by T-A (11.28%), A-T (11.01%) and C-T (11.01%), but G-C had the lowest percentage (4.61%) (Fig. 3B).

### MiRNA qPCR Validation and Spatial Expression

**Figure 4:**
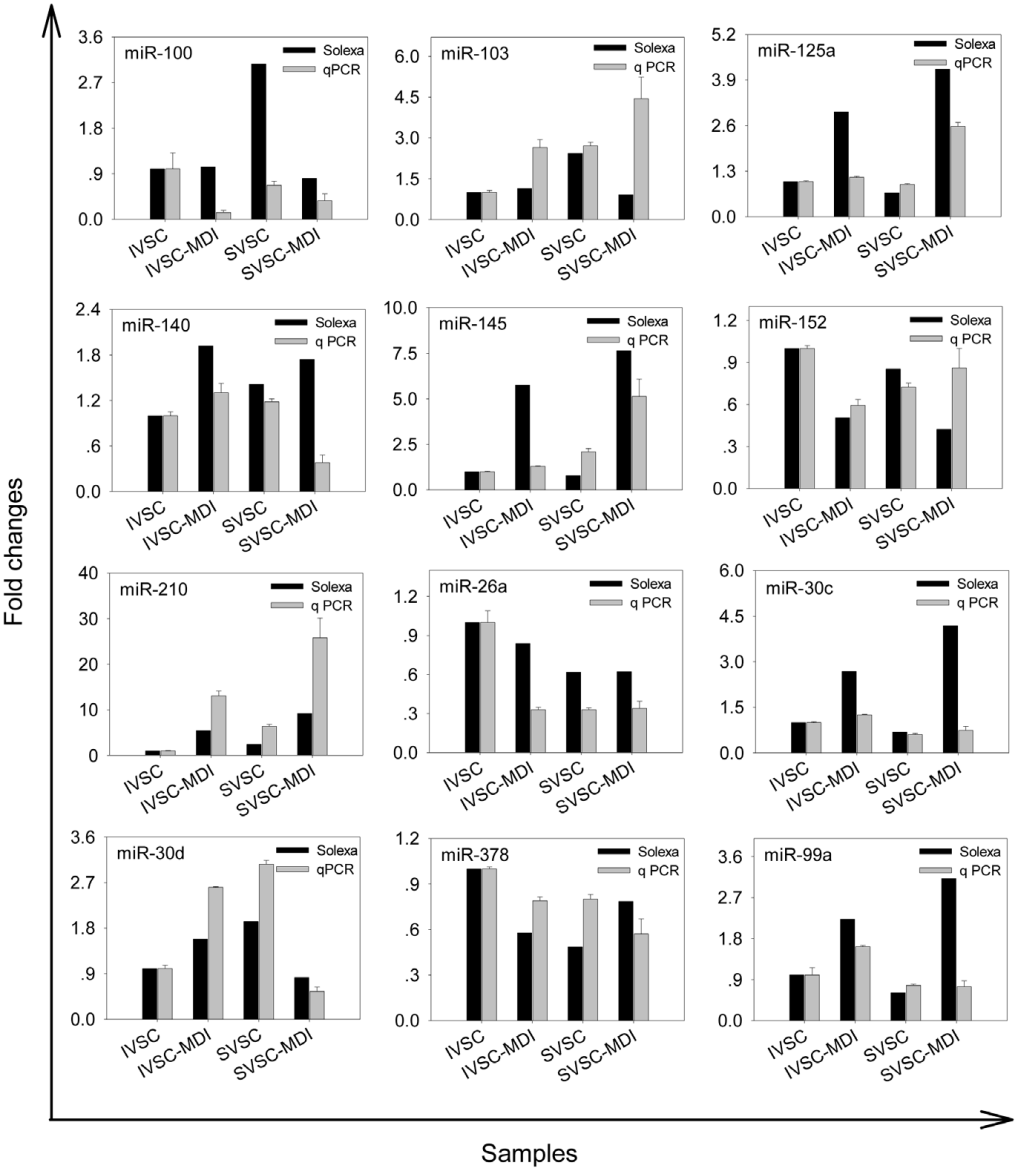
Validation of sequencing results with qPCR. The differing expressions of 12 miRNAs, as determined by sequencing, were validated by qPCR assays. For solexa sequencing, fold changes were calculated by comparing normalized reads of the same miRNA in IVSC-MDI and SVSC-MDI to IVSC and SVSC respectively. For qPCR experiment, fold changes were calculated the same as Figure1. The Y-axis here represented fold changes relative to IVSC.

**Figure 5:**
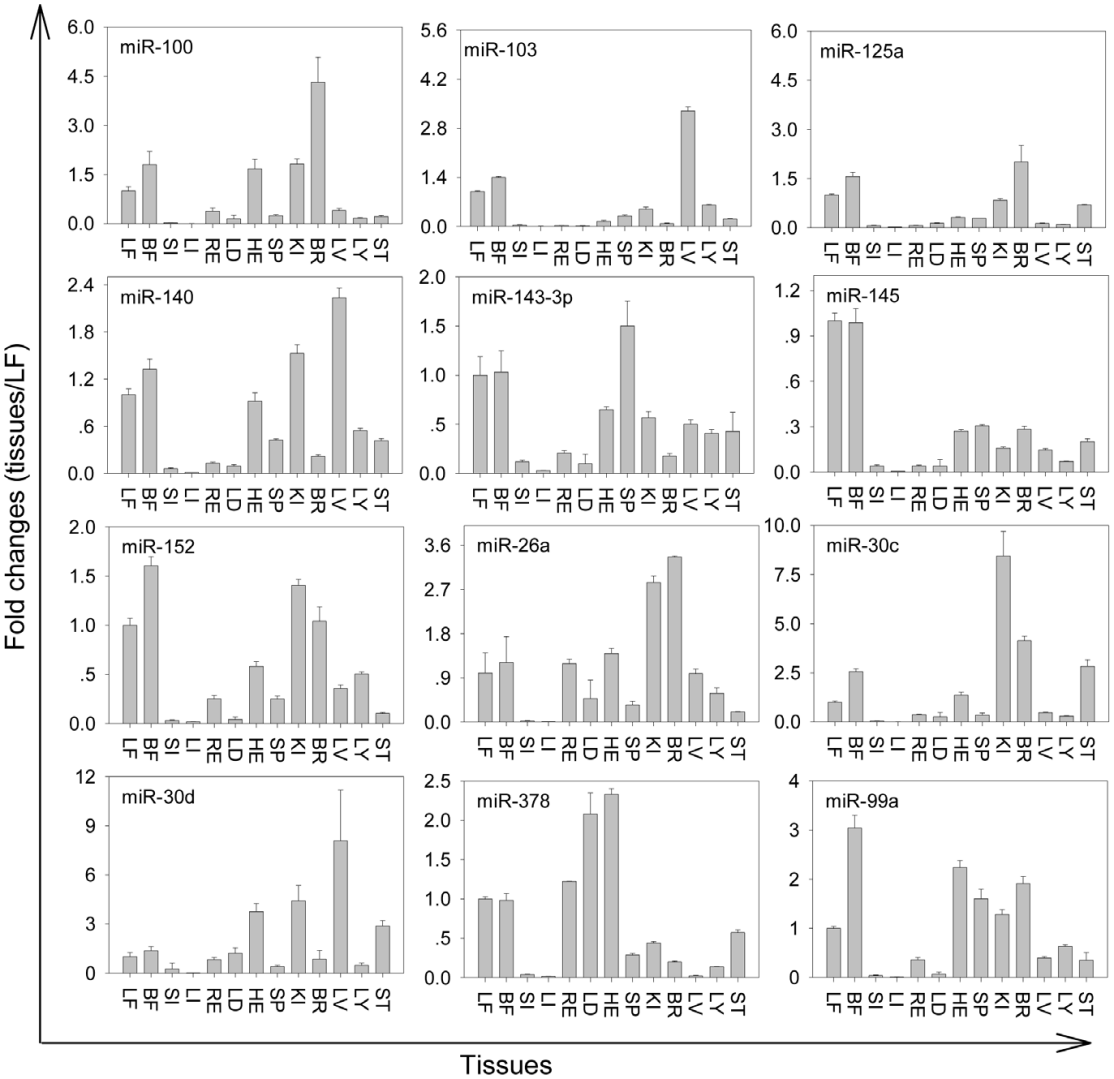
Spatial expressions of partial miRNAs identified during SVSC and IVSC differentiation. Relative expressions of 12 miRNAs in 13 porcine tissues were quantified by qPCR. The data were normalized to 18s rRNA, and were relative to their respective levels in leaf fat (LF). Data were expressed as mean±SD (n = 3). SI, small intestine; LI, large intestine; LF, leaf fat; BF, backfat; RE, retrahens; LD, longissimus dorsi muscle; HE, heart; SP, spleen; KI, kidney; BR, brain; LV, liver; LY, lymph; ST, stomach.

Swine miRNAs changed ≥2 fold or not were randomly chosen and validated with qPCR, and these miRNAs were expressed at different levels (Fig. 4). Among the 12 miRNAs listed, 9 had similar up- or down-regulated trends during both IVSC and SVSC differentiation, confirming our sequencing results. The expression patterns of the 12 miRNAs identified were quantified in 13 different tissues to determine whether miRNAs were expressed in a tissue-specific manner or found ubiquitously throughout the animal (Fig. 5). The 12 miRNAs were detected in all 13 tissues but were expressed differently. MiR-143-3p, miR-152, miR-378 and miR-103 were most highly expressed in spleen, backfat, heart and liver, respectively. MiR-145 was expressed considerably more highly in leaf fat and backfat. Expression levels of eleven miRNAs (except miR-30d) in leaf fat and backfat were moderate among the 13 tissues.

### MiRNAs Expression Changed during IVSC and SVSC Differentiation

**Figure 6:**
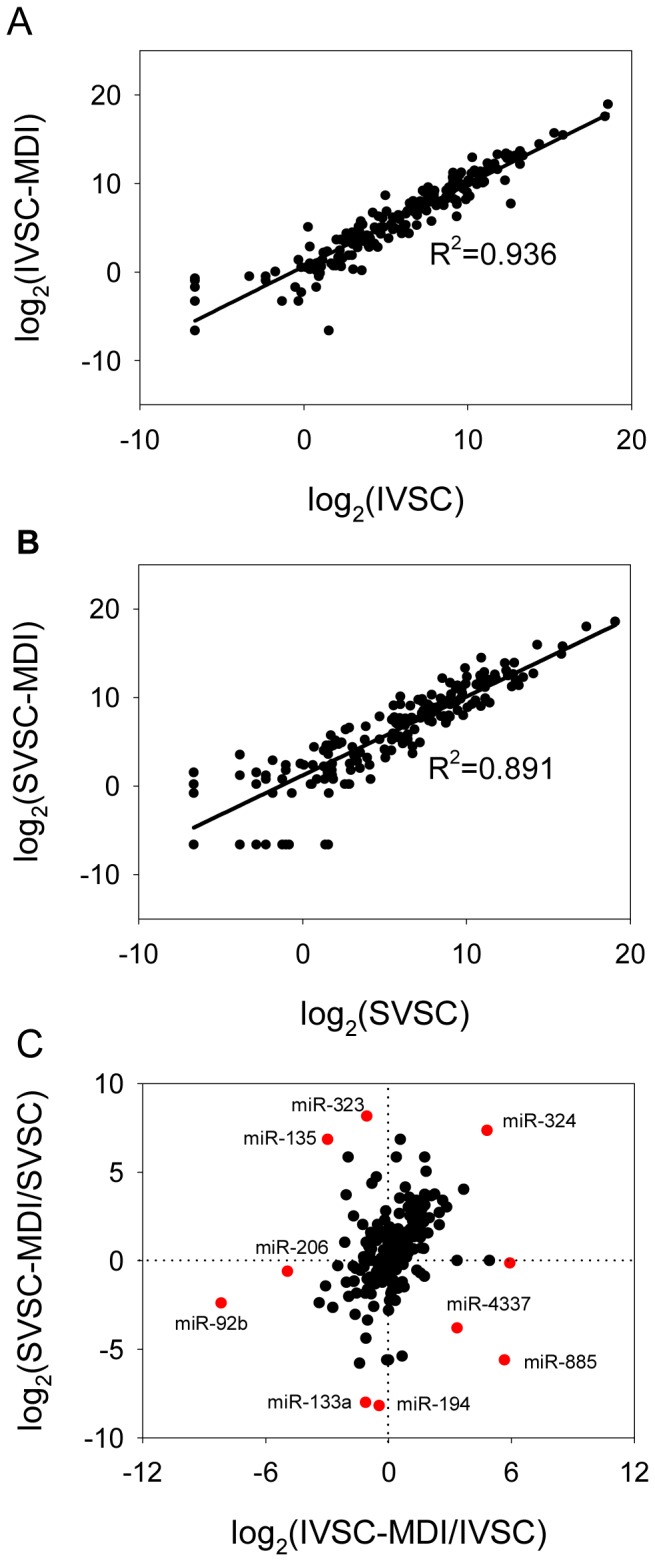
Correlation analysis of miRNAs expressed during IVSC and SVSC differentiation. Normalized expression values of swine miRNAs were calculated by log_2_ (A and B), and fold changes of miRNAs during IVSC and SVSC differentiation were also calculated by log_2_ (C), and the numbers marked in red indicating partial swine miRNAs that changed differently during IVSC and SVSC differentiation.

To determine whether most miRNAs expressed in similar line in the intensity scatter plot during IVSC and SVSC differentiation, correlation analysis was made. The miRNAs expression values in undifferentiated and differentiated VSC were significantly Pearson correlated (*p*<0.0001, R^2^ = 0.936 and 0.891 for IVSC and SVSC respectively, Fig. 6A and B). The fold changes of miRNAs during IVSC and SVSC differentiation were also significantly Pearson correlated (*p*<0.01, R^2^ = 0.314, Fig. 6C), but some miRNAs changed differently during IVSC and SVSC differentiation, including miR-135, miR-323, and miR-885 (Fig. 6C). To further identify the swine miRNAs that changed differently between IVSC and SVSC differentiation, those miRNAs changed ≥2 fold were chosen as candidates for cluster analysis, and the results are shown in Fig. S3. Thirty-three miRNAs were down-regulated and 50 were up-regulated in IVSC-MDI compared with IVSC. Fourty-two miRNAs were down-regulated and 77 were up-regulated during SVSC differentiation. Among these miRNAs, 17 were up-regulated and 37 were down-regulated uniformly during both IVSC and SVSC differentiation (such as miR-125a, miR-145, miR-210 and miR-92b); miR-4337 and miR-885 were up-regulated during IVSC differentiation but down-regulated during SVSC differentiation (Fig. 6C and Fig. S3A). Eight miRNAs showed the opposite trends (Fig. 6C and Fig. S3A). Fifty-five miRNAs changed significantly during only SVSC differentiation (such as miR-103, miR-365) (Fig. S3B), while 19 changed significantly during only IVSC differentiation (such as miR-27b, miR-206) (Fig. S3C). Therefore, 84 (61% of the 138 changed) miRNAs changed in different trends during IVSC and SVSC differentiation (Fig. S3D).

KEGG Orthology was applied to determine whether the miRNAs changed during SVSC and IVSC differentiation participate in adipogenesis (Table 1). In addition to the pathways mentioned above, other adipogenic pathways such as the adipocytokine, inositol phosphate metabolism, hedgehog and mTOR signaling pathway were also significantly changed during IVSC and SVSC differentiation. Apoptosis, cell cycle and ubiquitin-mediated proteolysis were also significantly enriched. The B cell receptor signaling pathway, cell adhesion molecules (CAMs), mRNA surveillance pathway, and RNA degradation were only enriched during SVSC differentiation, and dorso-ventral axis formation, ECM-receptor interaction, glycosphingolipid biosynthesis (lacto and neolacto series), N-glycan biosynthesis and VEGF signaling pathway were only enriched during IVSC differentiation.

**Table 1:** KEGG Orthology enriched for targets of changed miRNAs during IVSC and SVSC differentiation.

KEGG Orthology	IVSC-MDI/IVSC	SVSC-MDI/SVSC
Adherens junction	+	+
Adipocytokine signaling pathway	+	+
Apoptosis	+	+
Axon guidance	+	+
B cell receptor signaling pathway	−	+
Calcium signaling pathway	+	+
Cell adhesion molecules (CAMs)	−	+
Cell cycle	+	+
Dorso-ventral axis formation	+	−
ECM-receptor interaction	+	−
ErbB signaling pathway	+	+
Focal adhesion	+	+
Gap junction	+	+
Glycosphingolipid biosynthesis - lacto and neolacto series	+	−
GnRH signaling pathway	+	+
Hedgehog signaling pathway	+	+
Inositol phosphate metabolism	+	+
Insulin signaling pathway	+	+
MAPK signaling pathway	+	+
mRNA surveillance pathway	−	+
mTOR signaling pathway	+	+
Mucin type O-Glycan biosynthesis	−	+
Neurotrophin signaling pathway	+	+
N-Glycan biosynthesis	+	−
Notch signaling pathway	+	+
p53 signaling pathway	+	+
Pathways in cancer	+	+
Phosphatidylinositol signaling system	+	+
Regulation of actin cytoskeleton	+	+
RNA degradation	−	+
T cell receptor signaling pathway	+	+
TGF-beta signaling pathway	+	+
Tight junction	+	+
Type II diabetes mellitus	+	+
Ubiquitin mediated proteolysis	+	+
VEGF signaling pathway	+	−
Wnt signaling pathway	+	+

Note: +/−: Signaling pathway changed significantly or not during adipogenesis of IVSC and SVSC.

### MiRNAs Expressed in Fat-depot-specific Manner

**Figure 7:**
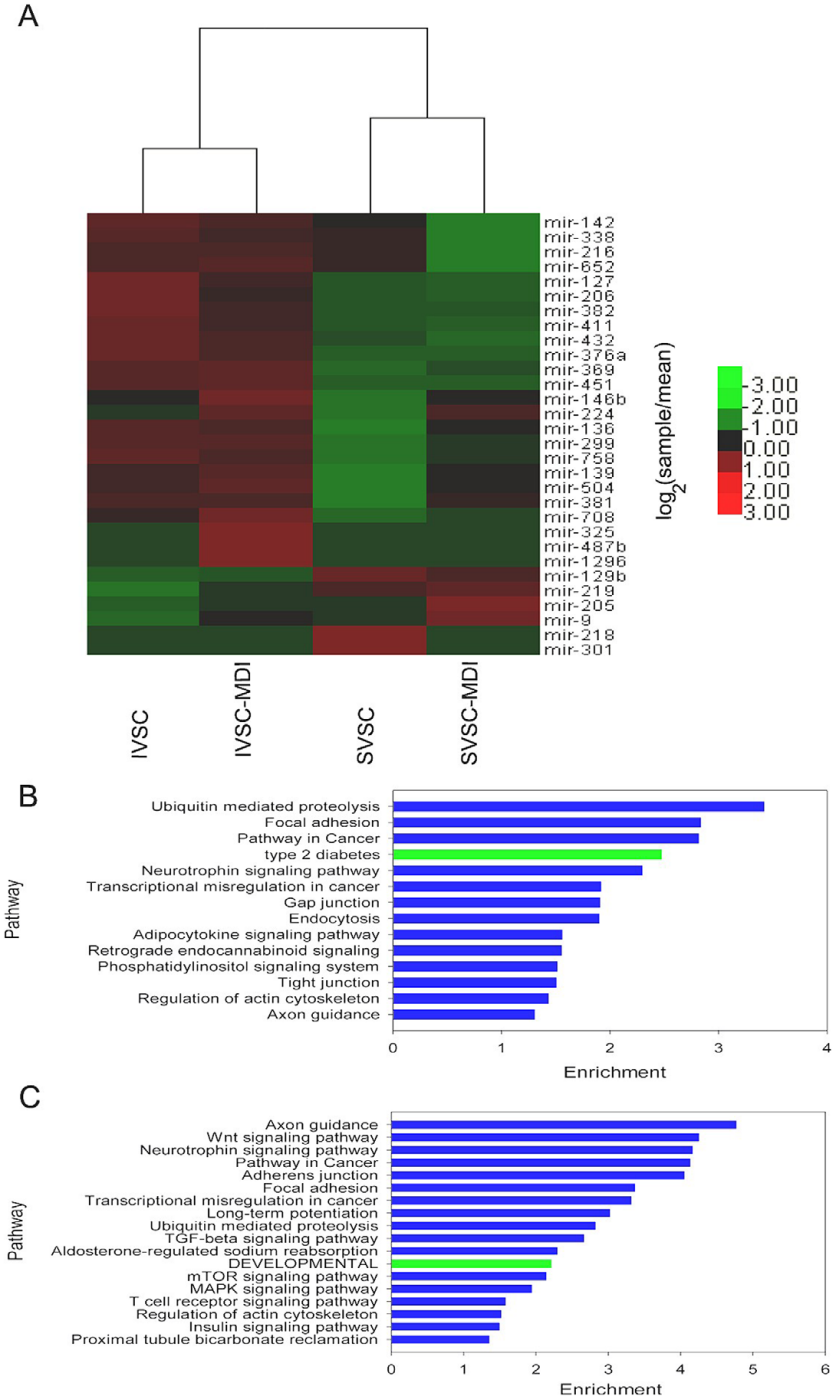
KEGG Orthology enriched for targets of the fat-depot-specific miRNAs. A: Cluster analysis of fat-depot-specific miRNAs. The normalized reads of a miRNA in any library was compared with its mean reads in the four libraries, then the data were calculated as log_2_. B: KEGG Orthology enriched for targets of the IMF specific miRNAs. C: KEGG Orthology enriched for targets of the subcutaneous fat specific miRNAs.

To determine whether miRNA expression is fat-depot specific, miRNA expression values between undifferentiated and differentiated adipocytes were compared. Seventy-two miRNAs were expressed ≥2 fold different between IVSC and SVSC. Sixty-nine miRNAs were expressed differently between IVSC-MDI and SVSC-MDI. Among the miRNAs, 36 differed between undifferentiated and differentiated VSC cells in both lines (Figure. S4A), 36 differed only between IVSC and SVSC and 33 only between IVSC-MDI and SVSC-MDI (Figure. S4B, C and D). Twenty miRNAs were more highly expressed in both IMF origin cells (such as miR-206, miR-369, miR-411) and 4 (miR-129b, miR-209, miR-205 and miR-9) were more highly expressed in both subcutaneous fat origin cells. Moreover, miR-325, miR-376a, miR-487b and miR-1296 were identified only in IMF origin cells, while miR-218 and miR-301 were identified only in SVSC. Overall, 30 miRNAs (24 in IMF origin cells and 6 in subcutaneous fat origin cells) may be expressed in a fat-depot-specific manner (Fig. 7A).

KEGG Orthology analysis was performed for the targets of fat-depot-specific miRNAs to determine their potential function (Fig. 7B and C). There were some pathways enriched significantly for targets of IMF and subcutaneous fat sepcific miRNAs simutaneously, such as axon guidance, pathway in cancer and ubiquitin mediated proteolysis (Fig. 7B and C). Developmental, insulin, mTOR, TGF-beta and wnt signaling pathway were enriched significantly only for targets of IMF sepcific miRNAs (Fig. 7B). Type 2 diabetes, adipocytokine, phosphatidylinositol and neurotrophin signaling pathway were enriched significantly only for targets of subcutaneous fat sepcific miRNAs (Fig. 7C).

### KEGG Orthology Analysis of Most Abundant miRNAs

**Figure 8:**
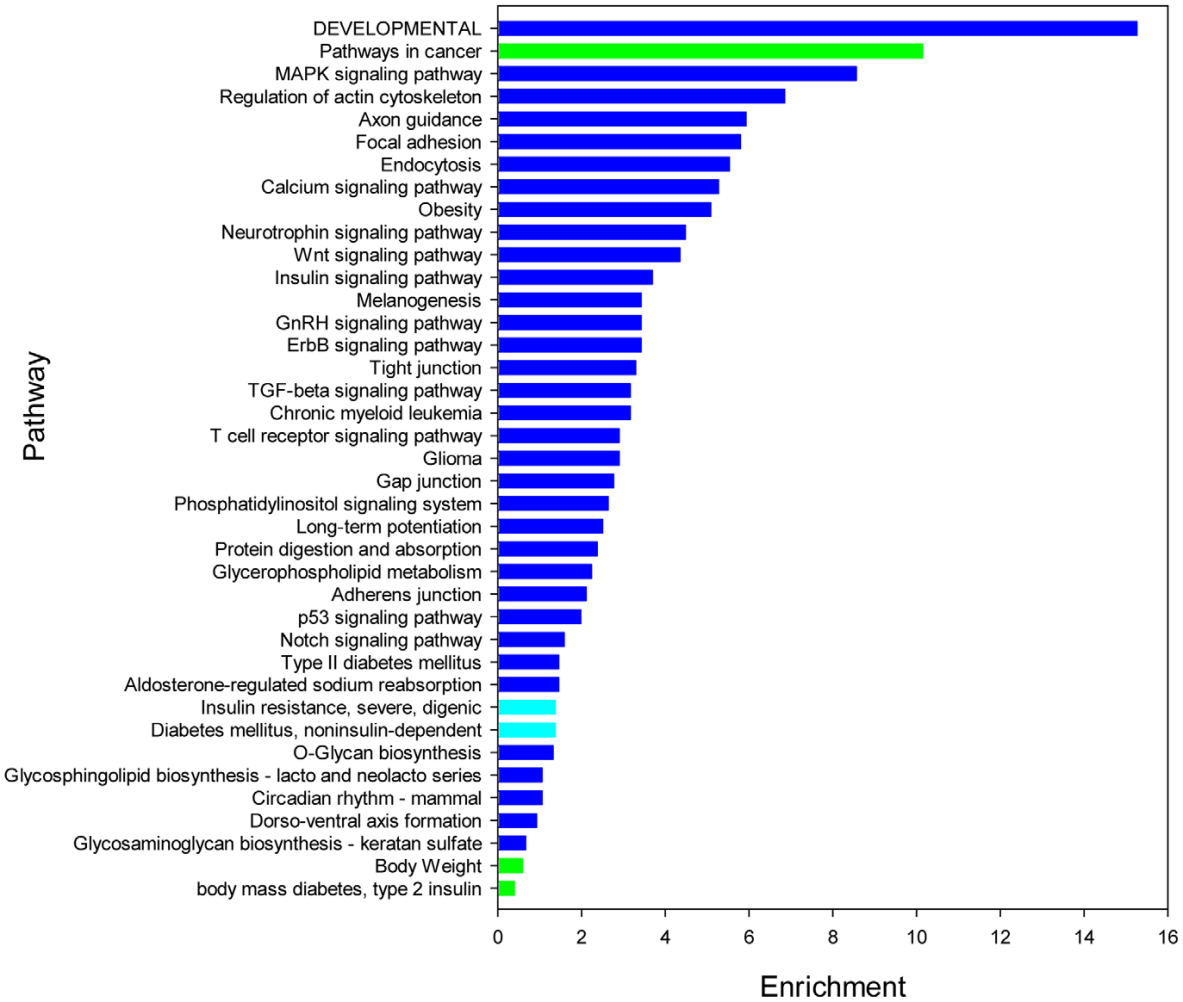
KEGG Orthology enriched for targets of the most abundant miRNAs. KEGG Orthology was done using targets of 21 most abundant miRNAs. Blue indicates KEGG pathway, green indicates GAD database, cyan indicates OMIM database.

To determine the potential functions of the identified miRNAs and considering the imperfect coverage of the pig genome, 21 most abundant miRNAs in the four libraries were selected for KEGG Orthology analysis. The abundances and fold changes of these miRNAs during IVSC and SVSC differentiation are shown in Table S1 and the KEGG Orthology results of 2607 target genes are shown in Fig. 8. The most abundant miRNAs participate most in developmental processes, including body weight, dorso-ventral axis formation, neutrophin signaling pathway. Some pathways involved in preadipocyte differentiation were also enriched, including the MAPK, wnt, insulin, TGF-beta and p53 signaling pathways. Obesity was significantly enriched, and obesity-related diseases (cancer), insulin resistance, and diabetes (insulin-dependent and non-insulin-dependent) were also observed. Glycan biosynthesis and lipid metabolism related pathways were significantly enriched.

### New Potential Porcine miRNAs

**Figure 9:**
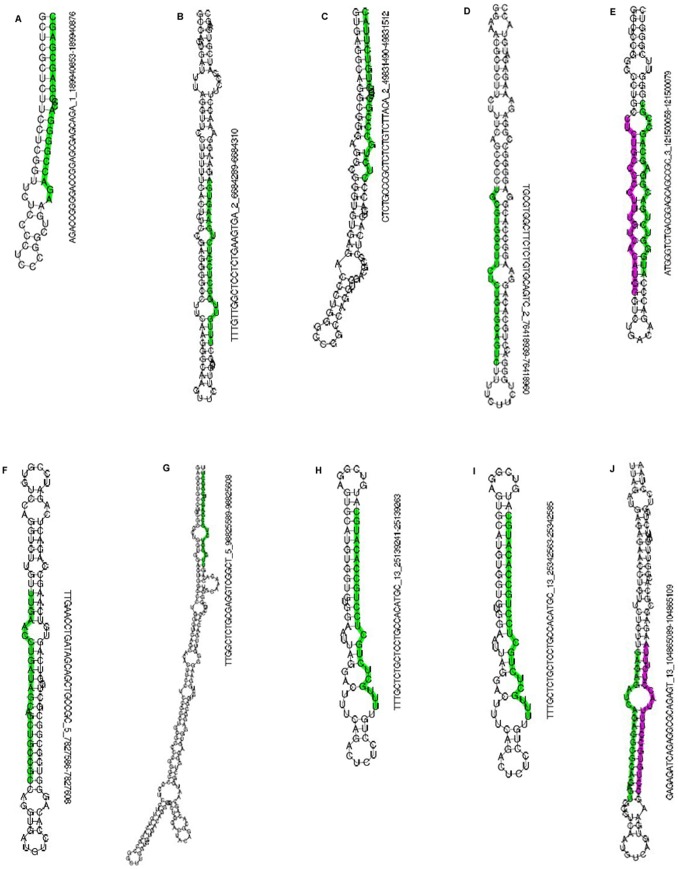
Hairpin structure of potential novel miRNAs. The stem-loop structures of 10 novel miRNAs predicted in the four libraries simultaneously were shown. The sequences in green represent potential mature miRNA sequences, and the sequences in pink represent homologous miRNA* sequences. The sequence and number on the right of each secondary structure are presented in the following model: genome sequence of potential mature miRNA_chromosome number_start position-end position.

Those sequences mapped to pig genome perfectly were selected as candidates for predicting new porcine miRNAs. According to the criteria mentioned in Materials and Methods, 280 unique sequences were identified by these methods and considered as putative new porcine miRNAs. The predicted stem-loop structures of 10 novel miRNAs predicted in the four libraries simultaneously are shown in Fig. 9, and information about all the putative novel miRNAs was compiled in Table S2. The novel miRNAs were distributed ubiquitously in all chromosomes and oriented from both the sense and antisense strands. The absolute sequencing abundances of these novel miRNAs ranged from 5 to 3316 (Table S2), and these sequences expressed with abundances <5 in other libraries or they may be fat-depot-specific (data not shown). Three of the novel miRNAs were detected with both stem-loop RT-PCR and the All-in-One™ miRNA qRT-PCR Detection system (data not shown). During IVSC and SVSC differentiation, 70 novel miRNAs were detected simutaneously, 95 and 123 miRNAs were detected only during IVSC and SVSC differentiation, respectively. What’s more, 76 and 112 novel miRNA sequences were identified only in IVSC and SVSC respectively, while 13 and 4 only in IVSC-MDI and SVSC-MDI respectively.

### MiRNAs that Regulate Adipogenesis Stimulated by Insulin

**Figure 10:**
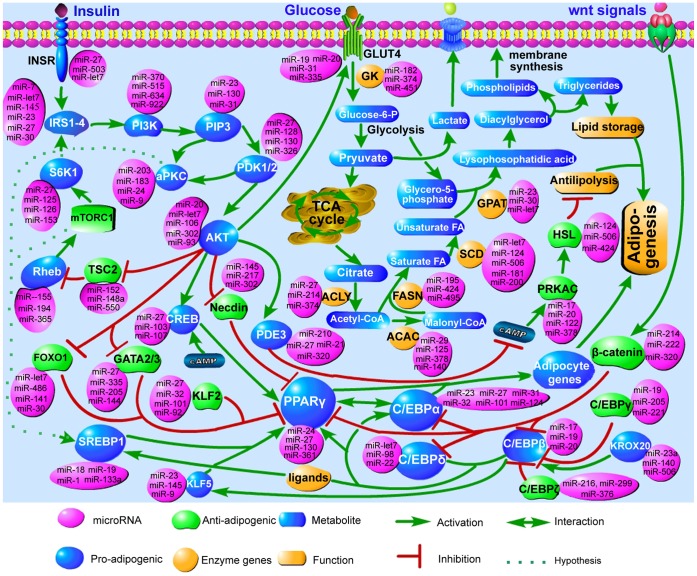
MiRNAs that regulate insulin-stimulated adipogenesis. A reverse search for miRNAs regulating mRNAs important in preadipocyte differentiation, lipogenesis and antilipolysis was performed. The relations between miRNAs and mRNAs were illustrated. *INSR*: insulin receptor; IRS1/2: insulin receptor substrate 1/2; *PI3K*: phosphoinositide-3-kinase, catalytic, polypeptide; *PIP3*: interaction protein for cytohesin exchange factors 1; *PDK1/2*: pyruvate dehydrogenase kinase, isozyme 1/2; *S6k1*: ribosomal protein S6 kinase, polypeptide 1; *aPKC*: protein kinase C, alpha; *SREBP1*: sterol regulatory element binding transcription factor 1; *AKT*: v-akt murine thymoma viral oncogene homolog; *TSC2*: tuberous sclerosis 2; *Rheb*: Ras homolog enriched in brain; *NEcdin*: necdin homolog (mouse); *CREB*: cAMP responsive element binding protein, *FOXO1*: RNA binding protein, fox-1 homolog 1; *FOXOA2*: forkhead box A2; *GATA2/3*: GATA binding protein 2/3; *PPARγ*: peroxisome proliferator-activated receptor gamma; *C/EBPα/β/γ/δ/ζ*: CCAAT/enhancer binding protein alpha/beta/gamma/delta/zeta; *KROX20*: early growth response 2, *KLF2/5*: Kruppel-like factor 2/5 (lung); *wnt10b*: wingless-type MMTV integration site family, member 10b; *β-catenin*: (cadherin-associated protein), beta 1; *Glut4*: solute carrier family 2 (facilitated glucose transporter), member 4; *GK*: glycerol kinase; *ACLY*: ATP citrate lyase; *ACAC*: acetyl-CoA carboxylase; *FASN*: fatty acid synthase; *SCD*: stearoyl-CoA desaturase (delta-9-desaturase); *GPAT2*: glycerol-3-phosphate acyltransferase 2, mitochondrial; *PDE3*: phosphodiesterase 3, cGMP-inhibited; *PRKAC*: protein kinase, cAMP-dependent, catalytic; *HSL*: lipase, hormone-sensitive.

Judging from the aspect of fat-depot specific miRNAs, insulin signaling pathway was different between the two cell lines, while judging from the aspect of changed miRNAs during adipogenesis, insulin signaling pathway was changed significantly during both IVSC and SVSC differentiation. To determine whether miRNAs regulate insulin-stimulated adipogenesis, a reverse search for miRNAs targeting genes regulated by insulin were performed and the relationship among them is illustrated in Fig. 10. Components in the insulin and *AKT* signaling pathways were regulated by miRNAs, such as the miR-27 family targeting *INSR*, *IRS1-4*, *PDK1/2* and *CREB*, miR-335 targeting *GATA2/3*, miR-let7 targeting *FOXO1/A2*. Enzymes related to glucose transport and triglyceride carbon skeleton production were targeted: *ACLY* by the miR-27 family, *FASN* by miR-495, miR-195 and miR-424. MRNAs related to antilipolysis were also regulated by miRNAs, *PDE3* was targeted by the miR-27 family, miR-21, and miR-210, *HSL* by miR-124, miR-506, miR-497, and miR-424. Further, markers during adipogenesis were also regulated by miRNAs, such as *PPARγ* was targeted by miR-27 family, *C/EBPα* by miR-23 family, and *C/EBPγ* by miR-222, miR-221, miR-205, and miR-19.

**Figure 11:**
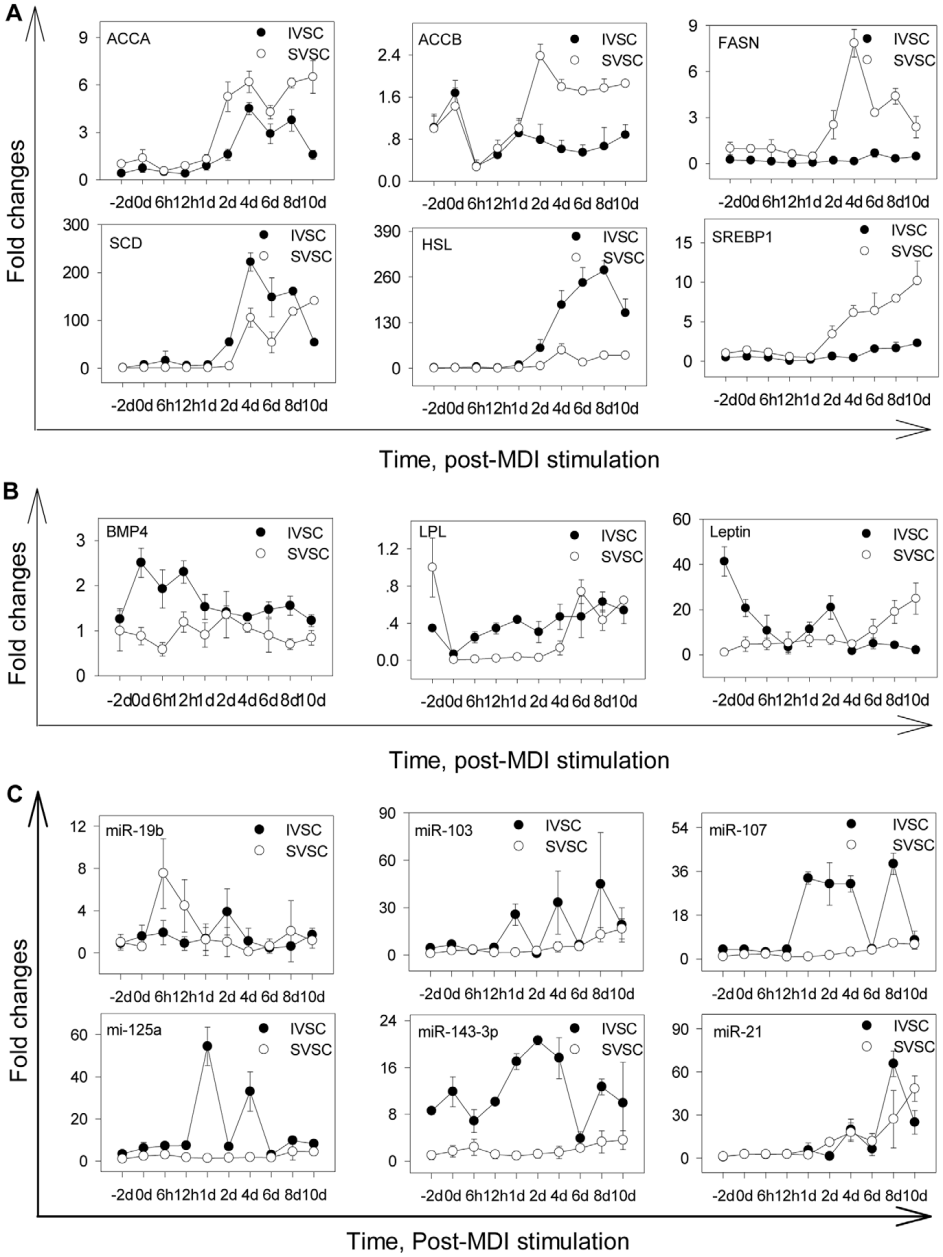
QPCR validation of mRNAs and miRNAs post insulin stimulation during IVSC and SVSC adipogenesis. Eleven insulin-stimulated mRNAs and 8 miRNA listed in Fig. 9 were quantified during IVSC and SVSC differentiation. Data analysis and replicates were the same as Figure 1. *Wnt10b*, *C/EBPα*, *C/EBPβ*, *PPARγ1*, *PPARγ2* are shown in Fig. 1M, and the others are shown in A. Some other mRNAs related to adipogenesis were selected and quantified by qPCR (B). MiR-27a, miR-27b and miR-210 are shown in Fig. 1N, and the others are shown in C. The adipogenic- related miR-143-3p was also quantified.

To study whether the mRNAs and miRNAs in Fig. 10 are regulated by insulin stimulation, 11 mRNAs and 9 miRNAs during IVSC and SVSC adipogenesis were quantified. Apart from wnt10b, *PPARγ*, *C/EBPα* and *C/EBPβ* mentioned above, some mRNAs were more highly expressed in SVSC than IVSC, such as *ACCA*, *ACCB*, *FASN* and *SREBP1*. They were up-regulated during IVSC and SVSC differentiation (Fig. 11A). However, *SCD* was expressed in the reverse pattern. There was mRNA expressed at lower levels in SVSC than IVSC, for example *HSL*. To determine whether other adipogenic mRNAs not included in Fig. 10 were also stimulated by insulin, three were chosen randomly. *BMP4* was more highly expressed during IVSC differentiation; *LPL* was up-regulated after MDI stimulation in both IVSC and SVSC; *leptin* was up-regulated in IVSC but down-regulated in SVSC (Fig. 11B). Apart from miR-27a, miR-27b and miR-210 mentioned above, miRNAs showed different expression pattern. MiR-19b was not changed during IVSC differentiation but was initially up-regulated during SVSC differentiation, returning to baseline level at day 10 (Fig. 11C). MiR-103, miR-107 and miR-125a were all more highly expressed during IVSC than SVSC differentiation (Fig. 11C). MiR-21 was up-regulated during IVSC and SVSC differentiation (Fig. 11C). MiR-143-3p was also up-regulated during SVSC differentiation, while two peaks were observed during IVSC differentiation (Fig. 11C).

## Discussion

### Similarities of miRNAs Regulate SVSC and IVSC Adipogenesis

Isolation of primary preadipocytes with high adipogenic potency is a key point in studying intramuscular and subcutaneous fat development *in vitro*. IVSC and SVSC isolated by our improved method could accumulate lipid droplets efficiently. Fifty-four (39% of the 138 changed) miRNAs changed in a similar manner during IVSC and SVSC differentiation. Previous studies have shown that some of these miRNAs are involved in the regulation of adipocyte differentiation. For example, miR-10b, miR-99a, miR-146b and miR-210 were up-regulated during SVSC and IVSC differentiation, while miR-130b was down-regulated. Similar results of these miRNAs were observed during 3T3-L1 differentiation [12]. MiR-10b, miR-99a miR-30a and miR-125b were up-regulated during SVSC and IVSC adipogenesis, and they were similarly expressed with pig backfat tissue development [10]. Moreover, miR-210 promoted adipogenesis [12]. However, some of the miRNAs, which did not change more than 2-fold in the present study, has been described regulating adipogenesis in other species. One example is miR-378. It was reprorted to increase the size of lipid droplets in ST2 mesenchymal precursor cells [15]. However, the expression values of miR-378 in differentiated porcine vascular stem cells were much lower than undifferentiated cells in the present study. Less than 2-fold change of miR-378 may be explained by its very high expression values in the libraries. Similarly, the expression of miR-378 in backfat of a 240-day-old pig was lower than in a 7-day-old piglet [10], and was down-regulated in animals with high backfat thickness [16]. In the same manner as miR-378, miR-221 and miR-222 had lower expressions in mature porcine VSC. In 9-day mature 3T3-L1 cells, expression levels of miR-221 and miR-222 were similar with preadipocytes [7]. The same manners of these two miRNAs were also observed during human primary subcutaneous preadipocytes differentiation [17]. However, miR-221 was up-regulated in mouse adipocytes from a model of insulin resistance and obesity [7], and in subcutaneous fat samples from obese patients compared with healthy and lean individuals [17]. These results indicate that miRNA expression and regulation may differ among species, and may differ with physiological status within a species. So, researchers should pay more attention to the physiological status of animals.

The predicted function of these miRNAs proved to be similar between IVSC and SVSC in adipogenesis. Developmental processes were significantly enriched by target genes of abundant miRNAs and changed miRNAs in both cell types in the current study. Adipocyte hypertrophy is an important part of adipose tissue development at the cellular level [18], and related pathways such as the mTOR [19], wnt [20], and adipocytokine [21] signaling pathways were also significantly enriched during both SVSC and IVSC differentiation. QPCR analysis of mRNAs in the above pathways, for example, *wnt10b* in the wnt signaling pathway and *leptin* in the adiocytokine signaling pathway, also indicated that these pathways were changed in the same manner during both IVSC and SVSC differentiation. Pig may be a better biomedical model organism than mouse for study human obesity and related diseases, and these abnormal states mainly marked by fat accumulation. Differentiation of adipocytes can mimic the development of obesity well, obesity and related diseases such as insulin resistance, type II diabetes mellitus and cancer were significantly enriched during IVSC and SVSC differentiation, these diseases are highly associated with total lipid content in skeletal muscle and subcutaneous fat depot [22]–[25]. Many miRNAs changed in a similar manner during IVSC and SVSC differentiation, suggesting that these miRNAs might have similar function during adipogenesis of different cell lines. The results may provide new miRNA targets for intramuscular and subcutaneous fat content, obesity and related diseases.

### Differences of miRNAs Regulate IVSC and SVSC Adipogenesis

IVSC accumulated triglycerides more slowly than SVSC during the 9 days of differentiation in the present study. Similar results have been reported in bovine and pig respectively [3], [4], [6]. This phenomenon was explained by Gondret *et al.* (2008) with a proteomic approach, proteins involved in anabolic and energy-yielding catabolic pathways were expressed at a lower level in intramuscular mature adipocytes than subcutaneous adipocytes, such as perilipin and VLCAD [5]. However, Chen *et al.* (2010) reported that intramuscular adipocytes possessed a higher capacity to accumulate lipid than visceral derived adipocytes during 16 days’ differentiation (although they were similar in the first 8 days), mainly because *PPARγ* and *C/EBPα* were expressed higher in the former cells [26]. The mechanisms that regulated IVSC and SVSC differentiation were not all studied, for adipocyte differentiation is regulated by a series of complex factors.

MiRNA is one of the important factors regulating preadipocyte differentiation. On the one hand, our sequencing data showed that expression levels of 55 and 19 miRNAs changed only during SVSC and IVSC differentiation respectively. Eight miRNAs were down-regulated during IVSC differentiation but up-regulated during SVSC differentiation, while 2 miRNAs showed the opposite trends. For example, miR-21 had similar expression levels in undifferentiated IVSC and SVSC, but it was up-regulated during SVSC differentiation before down-regulated at day 10 in IVSC. Similar with IVSC, human adipose tissue derived stem cells (ADSCs) up-regulated miR-21 expression firstly and then down-regulated it at day 8, miR-21 was confirmed to be the main miRNA that promotes ADSC adipogenesis [27]. Similar with human ADSCs [27], miR-143 was expressed at a much lower level than miR-21 in the present study; this may be because our IVSC and SVSC were ADSCs or vascular stem cells themselves [28], [29]. However, miR-143 was the most abundant miRNA in porcine backfat [10], showing that differences exist in primary adipocytes from adipose tissue, which contain many kinds of cells. MiR-103/107 and miR-100 were expressed with different abundance in the four libraries, but they were down-regulated in SVSC samples but not IVSC, but they all were expressed in a lower degree in subcutaneous samples from obese patients than lean individuals [17]. Similarly, in mouse subcutaneous primary adipocytes, miR-103/107 were expressed at low levels, but they were proved to promote aidpogenesis [30]. MiR-100 was down-regulated during 3T3-L1 differentiation [7], which was similar with our results. MiR-135, miR-133b, miR-184, miR-323 were down-regulated, and miR-196a, miR-34c, miR-4337, miR-885 were up-regulated during IVSC differentiation, while they presented the opposite expression pattern during SVSC differentiation. These results indicate that miRNAs may play different roles in IVSC and SVSC. On the other hand, 30 known miRNAs (24 and 6 in intramuscular and subcutaneous derived cells, respectively) and 218 potential miRNAs (95 and 123 in intramuscular and subcutaneous derived cells, respectively) were expressed in a fat depot-specific manner. Compared with previous studies about porcine miRNAs, 28 (10.0% in 280 new) miRNAs had been predicted [10], [31]–[34], but most of the potential miRNAs had very low reads. Interestingly, 76 and 112 potential novel miRNAs were predicted only in IVSC and SVSC, respectively, many more than in mature adipocytes, indicating that larger differences may exist between IVSC and SVSC as more miRNAs are identified, and these miRNAs may contribute to these differences.

### MiRNAs may Regulate mRNAs Stimulated by Insulin

Insulin signaling pathway was enriched significantly for targets of IMF specific miRNAs other than subcutaneous specific miRNAs. Therefore, it may be a target pathway to increase IMF content. In addition, insulin, which greatly stimulates adipogenesis, is a major differentiation-inducing hormone in our study. The adipogenic processes of SVSC and IVSC were regulated by a series of components in the insulin signaling cascade and *AKT* signaling pathway, lipogenesis and wnt signaling pathway (Fig. 10). *INSR*, *IRS1-4*, *PDK1/2*, *CREB*, and S6 kinase 1 (*S6K1*), which are involved in the insulin signaling cascade and the *AKT* signaling pathway, were targeted by the miR-27 family proved to inhibit adipogenesis [35]–[37]. In accordance with our prediction, the downstream *SREBP1*, which was more highly expressed during SVSC differentiation than IVSC, mediated insulin regulation of miR-1 and mir-133a [38]. Having similar expression pattern to *SREBP1*, *ACCA*, *ACCB* and *FASN* that involved in lipogenesis were expressed higher during SVSC adipogenesis than IVSC, and they were targeted by miRNAs such as miR-140, miR-195 and miR-424. Wnt signaling has been reported to be regulated by miRNAs during adipogenesis [12]. In the present study, *wnt10b*, which was down-regulated during both IVSC and SVSC differentiation, was targeted by miR-148 and miR-152 (data not shown). Adipogenic markers were regulated by the mRNAs mentioned above together with the miRNAs. In accordance with our prediction, miR-27 has been reported to target *PPARγ* and *RXRα* directly [36], [37]. Therefore, miRNAs may play their regulating roles through targeting genes stimulated by insulin. Though single mRNA and miRNA may be stimulated differently by insulin during SVSC and IVSC differentiation, similar pathway were changed in the process. So our further study should focus on single mRNA or miRNA. The present solexa deep sequencing results were validated by qPCR experiment based on three pigs, but the cells for deep sequencing were just isolated from one pig, the results may need to be validated with more biological replicates.

### Conclusion

This is a novel study comparing miRNAomes of IVSC and SVSC during differentiation. MiRNAs regulate IVSC and SVSC differentiation by targeting similar pathways related to adipogenesis and obesity. Fat-depot-specific miRNAs may be used as biomarkers or targets for enhancing intramuscular content. Identifying more porcine miRNAs and uncovering their function during IVSC and SVSC differentiation needs further investigation, not only to further our understanding of how these miRNAs regulate marbling in agricultural animals globally, but also to identify therapy targets for obesity.

## Materials and Methods

### Ethics Statement

Animals were cared for according to the guidelines developed by the China Council on Animal Care and procedures approved by the Animal Care and Use Committee of Guangdong Province, China. The approval ID or permit numbers have been supplied in a former report [39].

### Isolation and Differentiation of IVSC and SVSC

To establish a differentiation model of intramuscular vascular stem cells (IVSC) and subcutaneous vascular stem cells (SVSC), differentiation potency of the cells isolated were examined. Three healthy boar pigs (Landrace), no more than 24 h old, were selected from a high-health swine breeding center. The dorsal longissimus dorsi and subcutaneous fat tissue were dissected, primary IVSC and SVSC cells were isolated, plated and induced to differentiation according to Text S1. Cells cultured in GM and MDI at day 9 were lysed and triglycerides contents were determined using a Triglyceride Kit (Biosino Bio-Technology and Science Inc., Beijing, China) according to the manufacturer’s protocol. Total protein of the cell lysate was quantified with the Bradford Protein Assay Kit (Beyotime) according to the manufacturer’s instructions. Adipogenic-related mRNAs (*wnt10b*, *C/EBPβ*, *PPARγ1*, *PPARγ2*, *C/EBPα*, *GPDH* and *ap2*/*FABP4*) and miRNAs (miR-27a, miR-27b and miR-210) of the above mentioned time points during IVSC and SVSC differentiation were tested with qPCR; C/EBPα protein was determined by western blotting.

### QPCR Experiments

All qPCR experiments were conducted as follows: (1) Total RNA was extracted using TRIzol reagent (Invitrogen) according to the manufacturer’s instructions. (2) First-strand cDNA of mRNAs and miRNAs were obtained using the Reverse Transcription System (Promega) and All-in One™ First-Stand cDNA Synthesis Kit (GeneCopoeia) according to the manufacturer’s instructions, respectively. (3) QPCR was applied using SYBR Premier Dimer Eraser™ (TaKaRa) according to the manufacturer’s instructions on a LightCycler480 (Roche), relative quantification (△△Ct) method was used to analyze the data. For qPCR experiments, three boar pigs of the same age mentioned above were used, and triplicate of cells from each pig were used. Endogenous GAPDH mRNA was used as reference for mRNA and miRNA in cells as mentioned previously [40], [41], for U6, 18S rRNA and 5S rRNA for miRNA were detected not stably expressed during their differentiation compared with *GAPDH*. For spatial expression experiments, 13 tissues, including small intestine, large intestine, leaf fat, backfat, retrahens, longissimus dorsi muscle, heart, spleen, kidney, brain, liver, lymph, and stomach, were obtained from three 180-days old boar pigs. 18S rRNA was used as reference for miRNAs in tissues. The primers of mRNAs and miRNAs were all listed in Table S3 and Table S4, respectively.

### Western Blotting

Cells at different days after MDI stimulation were lysed in cell lysis buffer (Beyotime) containing 1 mM phenylmethylsulfonyl ﬂuoride, and the protein concentration was quantified as mentioned above. Equal amounts of total cellular protein were fractionated by 12% (w/v) SDS/PAGE and electronically transferred to 0.2 µm PVDF membrane (Bio-Rad). The membrane was rinsed with TBS-Tween20 (TBST), blocked for 2 h in TBST containing 5% (w/v) skimmed milk, and incubated with primary antibody for 1 h. The membrane was washed with TBST and incubated for 1 h with secondary antibody conjugated to HRP. Blots were visualized using an ECL detection kit (Thermo Scientific). The primary antibody for C/EBPα was rabbit anti-C/EBPα (#2295, Cell Signaling Technology), and the secondary antibody was HRP-conjugated goat anti-rabbit IgG. The primary antibody for GAPDH (sc-59540, Santa Cruz Biotechnology) was mouse anti-GAPDH, and the secondary antibody was HRP-conjugated goat anti-mouse IgG.

### Total RNA Isolation, Small RNA Libraries Construction and Sequencing

Undifferentiated (d0) and differentiated (d9) IVSC and SVSC cells were prepared as detailed in Fig. 1A (named IVSC, IVSC-MDI, SVSC and SVSC-MDI, respectively) from a boar pig. Total RNA was isolated using the methods mentioned above. Total RNA quality was controlled using Agilent Technologies 2100 Bioanalyzer (Agilent Technologies, Santa Clara, CA) with an RNA Integrity Number (RIN) value >7 and 28S/18S>2.

For Solexa sequencing, the same amount of total RNAs isolated from cells were pooled, and small RNAs were prepared as follows: 10 µg total RNA of each sample was size-fractionated by 15% agarose gel electrophoresis and RNA fragments of 16–35nt length were collected. The first reaction was to ligate small RNA 3′ adapters to the isolated small RNA, followed by 5′ adapter ligation (Illumina, CA, USA). The small RNA ligated with 3′ and 5′ adapters was size-fractionated on a 10% agarose gel, and the 70–90nt fraction (small RNA + adaptors) was isolated. Reverse transcription, followed by PCR at 12 cycles to enrich the fragments with ligated adapters at each end, was used to create cDNA constructs. PCR forward and reverse primers annealed to the ends of the adapters. The amplification products were purified using 6% Novex TBE PAGE and small RNA libraries were validated using the Agilent Technologies 2100 Bioanalyzer. The well validated libraries were directly used for cluster generation and 36 cycles of sequencing analysis using the Illumina Cluster Station and 1G Genome Analyzer, following the manufacturer’s protocols.

### Computational Analysis of Deep Sequenced Small RNA

Open source Firecrest and Bustard applications were applied to extract sequencing reads from the image files generated by the Illumina 1G Genome Analyzer. Adapter sequences, sequences with p_error_ (p_error_ = 10^Q/−10^, Q represents quality score) values ≥0.05, and ambiguous nucleotides (maximum two nucleotides allowed) were removed, and sequences between 15 and 55nt were first mapped to swine sequences in the miRBase. Sequences containing up to two mismatches (or >90% identity) with swine miRNAs were considered as putative swine miRNAs and assigned the same name as that of the top match. Chromosome locations of the sequencing reads without adaptors were determined using short oligonucleotide analysis package (SOAP) software (http://soap.genomics.org.cn) by mapping the sequences to the pig genome. The classes of small RNAs identified were classified with Sus_scrofa.Sscrofa 9.63 (ftp://ftp.ensembl.org/pub/release-63/gtf/sus_scrofa/Sus_scrofa.Sscrofa9.63.gtf.gz). Sequences with more than 5 reads and a single nucleotide mismatch to pig genome were chosen; the percentage of each of 12 nucleotide substitution types in any chromosome and in mitochondria (MT) was calculated in each library; and the results were also integrated as a whole. SPSS software (16.0) was used for correlation analysis of the nucleotide substitution results. The unannotated deep sequencing reads were also mapped to the pig genome, with those that could map to >100 loci being removed to avoid repeat sequences. The remaining sequences were input into the UEA sRNA Toolkit together with porcine genome sequences, and the sequences mentioned above were mapped to the genome with the miRCat tool. MiRCat was used to identify genomic regions covered with sequences (small RNA loci) that had an abundance at least five, the following criteria were employed to select likely miRNA candidates: (1) Loci must contain no more than four non-overlapping sRNAs; (2) Each sRNA in a locus must be no more than 200nt away from its closest neighbor; (3) At least 90% of sRNAs in a locus must have the same orientation. The sRNAs with the most abundant read within a locus were selected as the likely miRNA, and also extracted flanking sequences surrounding this sRNA from the genome using varying window lengths. The secondary structure of each sequence window was predicted with RNAfold. The following features were used to classify miRNAs: (1) number of consecutive mismatches between miRNA and miRNA* must be no more than 3; (2) number of paired nucleotides between the miRNA and the miRNA* must be at least 17 of the 25 nucleotides centered around the miRNA; (3) the hairpin must be at least 50nt in length; (4) the percentage of paired bases in the hairpin must be at least 50% of base-pairs in the hairpin; (5) sequence homology to known mammalian miRNAs was used as a criterion. The hairpin with the lowest minimum free energy (MFE) from the sequence windows was chosen as the precursor miRNA (pre-miRNA) candidate and tested using Randfold, with a default cutoff of 0.1.

### Differential Expression Analysis

To determine the change of miRNAs during IVSC and SVSC differentiation, the expression levels of miRNAs were normalized to per million reads. If the normalized expression value of a miRNA was 0, then 0.01 was used to modify its expression. The fold changes of a miRNA during IVSC and SVSC differentiation was calculated by comparing its normalized expression values in IVSC-MDI and SVSC-MDI to that in IVSC and SVSC respectively. Any miRNAs with ≥2-fold changes were selected for differential and bioinformatical analysis.

### MicroRNA Targets Prediction and KEGG Orthology Analysis

Since the current swine miRNA assembly has imperfect coverage and in view of the functional conservation of miRNAs, the 15 most abundant miRNAs in IVSC, IVSC-MDI, SVSC and SVSC-MDI respectively were selected to predict their possible biological functions. After integration, there were 22 swine miRNAs in all. The targets of 21 (not including miR-206) swine miRNAs were predicted using the online database mirecords based on the sequences of the miRNAs (http://mirecords.biolead.org/), and the targets predicted in at least five databases integrated in mirecords were chosen. The KEGG Orthology was analyzed using the online database http://kobas.cbi.pku.edu.cn, KEGG PATHWAY, KEGG DISEASE, GAD, FunDO, OMIN and NHGRI were selected, and the pathways giving statistically significant values (*p*<0.05) were chosen. KEGG Orthology analysis was also conducted using miRNAs changed during IVSC and SVSC to determine whether the miRNAs functioned differently in different fat depots.

To determine whether any of the miRNAs participated in swine IVSC and SVSC differentiation, a reverse search for miRNAs regulating mRNAs important in preadipocyte differentiation, lipogenesis and antilipolysis was performed, such as *PPARγ* and *C/EBPα*. Targetscan (http://www.targetscan.org/) and microrna.org (www.microrna.org/microrna/getGeneForm.do) databases were used. The results showed a high probability of preferential conservation in Targetscan or good mirSVR scores and phastcons score in microrna.org were chosen.

## Supporting Information

Figure S1 **Saturation plots of small RNA libraries.** Saturation plots were created from one of the 4 small RNA libraries to indicate that the composition of small RNAs would remain constant as more reads were added. Total reads were divided into 6 equal parts, and the number of unique small RNA (A); the percentages of annotated small RNA, annotated read and miRbase found (B) were calculated respectively. M represents million.
(TIF)

Figure S2 **Small RNA length distribution during IVSC and SVSC differentiation.** Small RNA reads among 15–35nt are summarized, and the distribution curves of SVSC, SVSC-MDI, IVSC, IVSC-MDI and average of the 4 libraries are also calculated.
(TIF)

Figure S3 **Cluster analysis of differently expressed miRNAs during VSC differentiation.** The swine miRNAs that changed ≥2-fold during IVSC or and SVSC differentiation were chosen as candidates for cluster analysis. The normalized reads of a miRNA in any library was compared with its mean reads in the four libraries, then the data were calculated as log_2_. A shows the miRNAs changed ≥2-fold during both IVSC and SVSC differentiation; B shows the miRNAs changed ≥2-fold only during SVSC differentiation; C shows the miRNAs changed ≥2-fold only during IVSC differentiation, D shows the numbers of miRNAs listed in A, B and C, and there were138 miRNAs in all.
(TIF)

Figure S4 **MiRNAs expressed differently in undifferentiated VSC and differentiated VSC.** Expression values of miRNAs in IVSC, SVSC, IVSC-MDI and SVSC-MDI libraries were normalized. Fold changes ≥2-fold among VSC and differentiated VSC were chosen, and the fold changes were calculated as log_2_. A represents those miRNAs different in both undifferentiated VSC and differentiated VSC; B represents those miRNAs only different between IVSC and SVSC; C represents the miRNAs only different between IVSC-MDI and SVSC-MDI, D represents the numbers of miRNAs listed in A, B and C.
(TIF)

Table S1 **Expression values and fold changes of the most abundant miRNAs in undifferentiated and differentiated VSC.**
(DOC)

Table S2 **Sequences and chromosome positions of all potential novel miRNAs.** Considering the multiple information presented, the results were collated in an Excel file. The first 4 columns provide the chromosome location of potential miRNAs; the E column gives the abundances of potential miRNAs in samples, and abundances are ranked in accordance with the O column, which details the samples containing the predicted miRNAs; the F and G columns show the sequences and length of the potential miRNAs; the H column has the number of the miRNA hits in the genome sequence; the I and J column show the length and GC percent of the hairpin structure; the K and L columns contain the minimum free energy and adjusted minimum free energy; the M column details the Randfold *p*-value; the N column shows the predicted miRNA* sequence(s), if any, along with abundance in input dataset shown in brackets and the ranks of abundances, which are also in accordance with column O. P column showed those novel porcine miRNAs predicted in previous reports. Q column showed those miRNA* predicted in previous reports. R and S columns showed sequence homology of novel miRNAs to known mammalian miRNAs.
(XLS)

Table S3 **QPCR primers of mRNAs.**
(DOC)

Table S4 **QPCR primers of miRNAs.**
(DOC)

Text S1 **The procedures for isolation, plating and differentiation of VSC cells.**
(DOC)
