# Supplementary figures and images for: MicroRNAome Comparison between Intramuscular and Subcutaneous Vascular Stem Cell Adipogenesis
Source: PLoS One. 2012 Sep 20;7(9):e45410. doi: 10.1371/journal.pone.0045410 (PMC3447870; doi:10.1371/journal.pone.0045410)

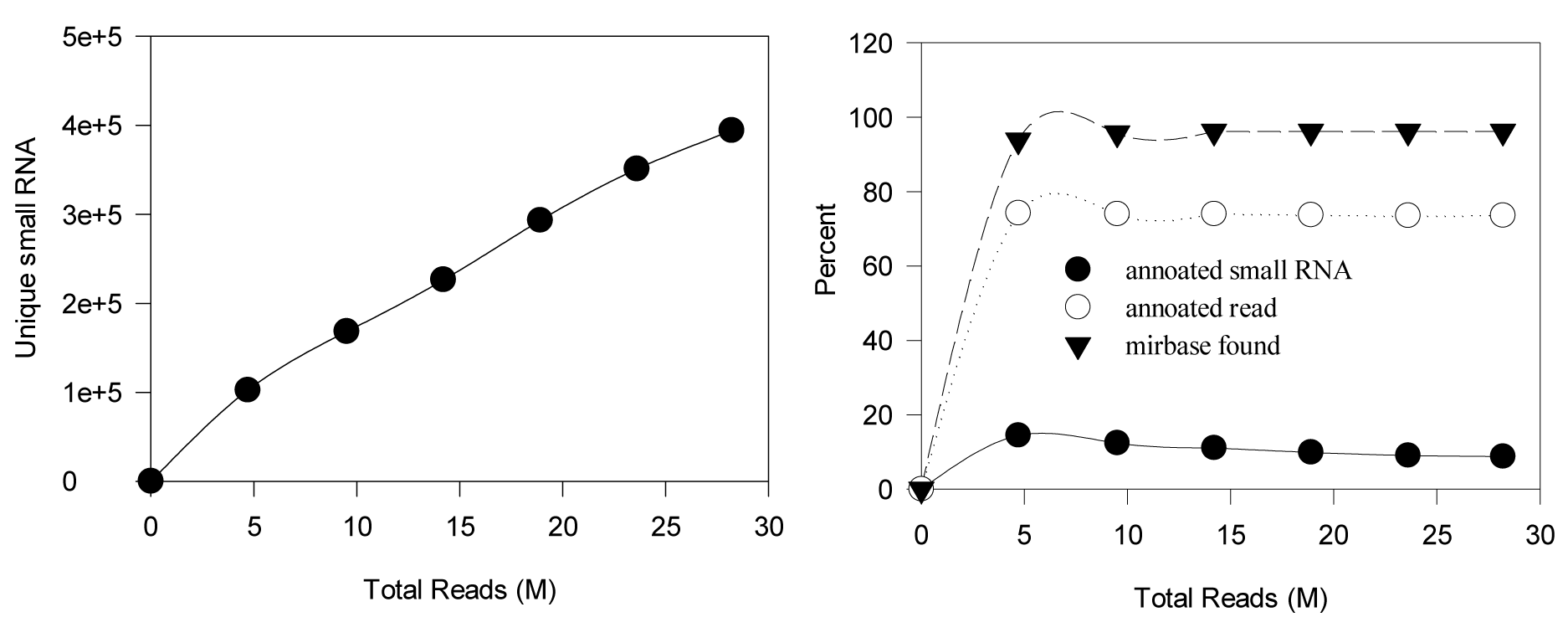

Supplement: Figure S1 — Saturation plots of small RNA libraries. Saturation plots were created from one of the 4 small RNA libraries to indicate that the composition of small RNAs would remain constant as more reads were added. Total reads were divided into 6 equal parts, and the number of unique small RNA (A); the percentages of annotated small RNA, annotated read and miRbase found (B) were calculated respectively. M represents million. (TIF) [file pone.0045410.s001.tif]

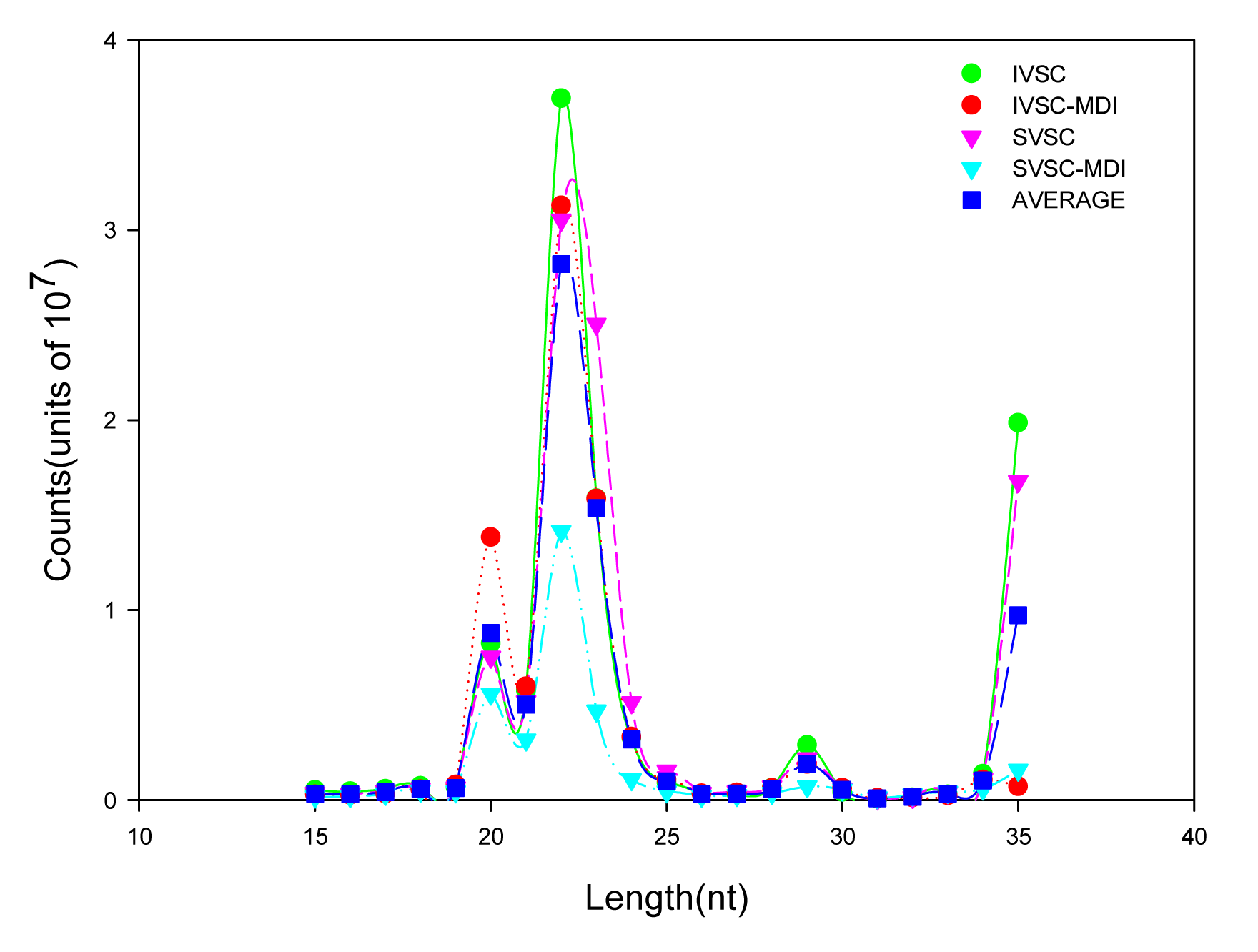

Supplement: Figure S2 — Small RNA length distribution during IVSC and SVSC differentiation. Small RNA reads among 15–35nt are summarized, and the distribution curves of SVSC, SVSC-MDI, IVSC, IVSC-MDI and average of the 4 libraries are also calculated. (TIF) [file pone.0045410.s002.tif]

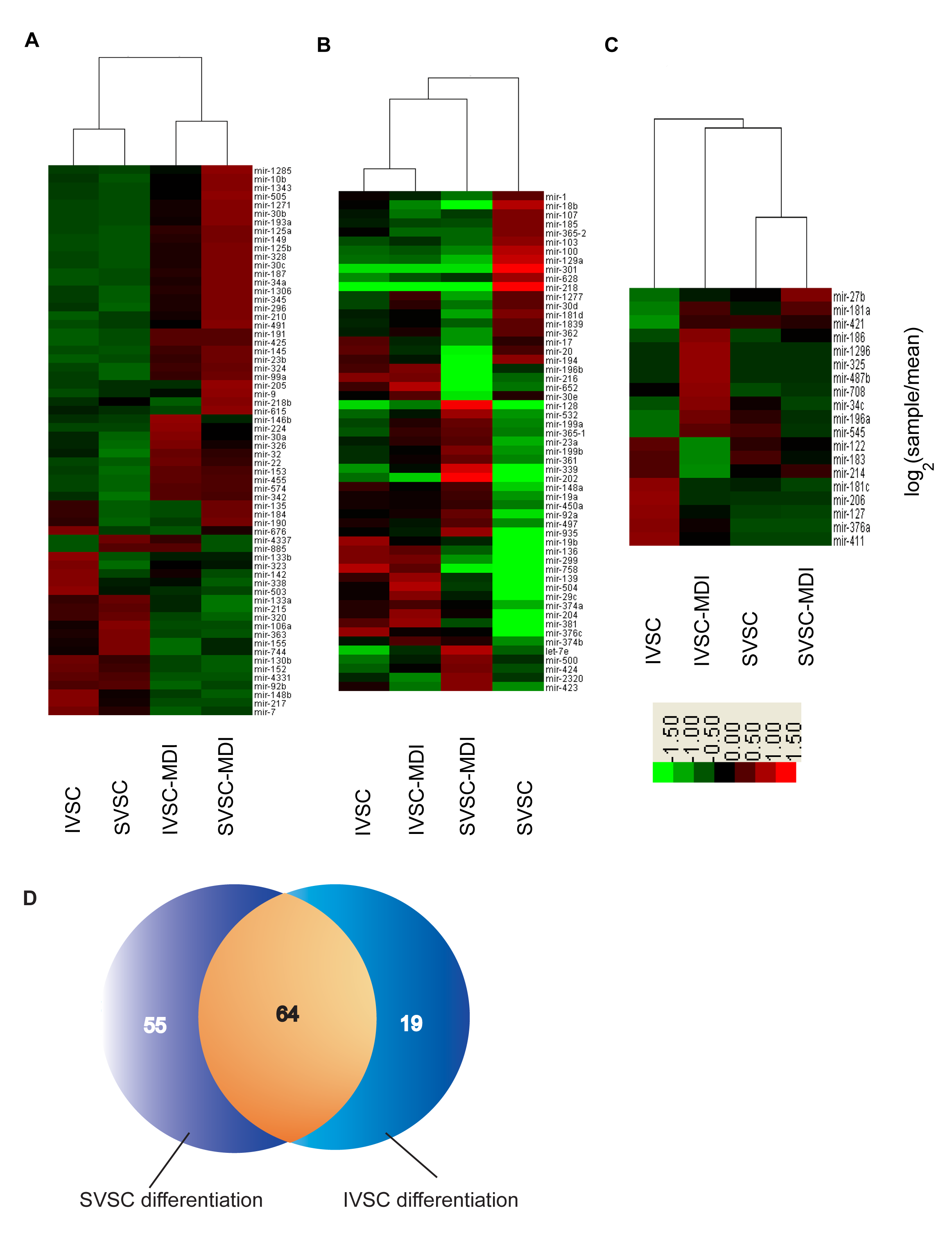

Supplement: Figure S3 — Cluster analysis of differently expressed miRNAs during VSC differentiation. The swine miRNAs that changed ≥2-fold during IVSC or and SVSC differentiation were chosen as candidates for cluster analysis. The normalized reads of a miRNA in any library was compared with its mean reads in the four libraries, then the data were calculated as log2. A shows the miRNAs changed ≥2-fold during both IVSC and SVSC differentiation; B shows the miRNAs changed ≥2-fold only during SVSC differentiation; C shows the miRNAs changed ≥2-fold only during IVSC differentiation, D shows the numbers of miRNAs listed in A, B and C, and there were138 miRNAs in all. (TIF) [file pone.0045410.s003.tif]

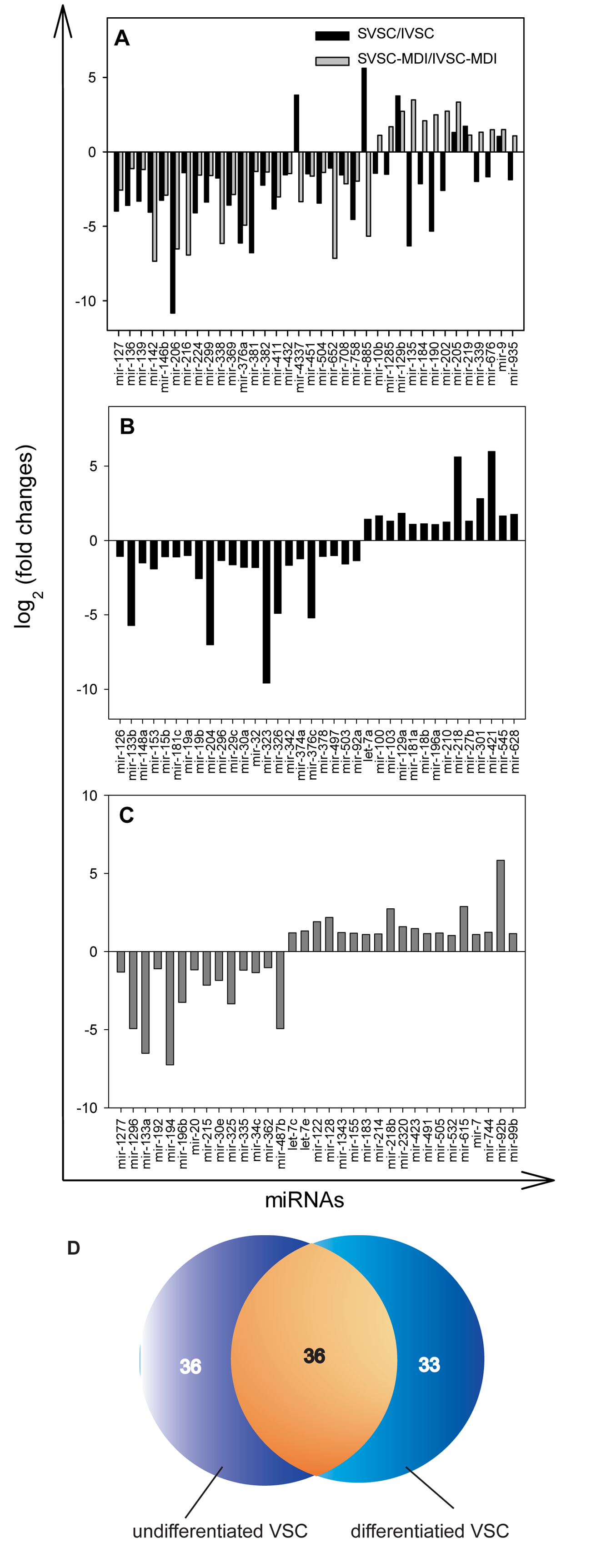

Supplement: Figure S4 — MiRNAs expressed differently in undifferentiated VSC and differentiated VSC. Expression values of miRNAs in IVSC, SVSC, IVSC-MDI and SVSC-MDI libraries were normalized. Fold changes ≥2-fold among VSC and differentiated VSC were chosen, and the fold changes were calculated as log2. A represents those miRNAs different in both undifferentiated VSC and differentiated VSC; B represents those miRNAs only different between IVSC and SVSC; C represents the miRNAs only different between IVSC-MDI and SVSC-MDI, D represents the numbers of miRNAs listed in A, B and C. (TIF) [file pone.0045410.s004.tif]
