# Supplementary material for: MicroRNAome Comparison between Intramuscular and Subcutaneous Vascular Stem Cell Adipogenesis
Source: PLoS One. 2012 Sep 20;7(9):e45410. doi: 10.1371/journal.pone.0045410 (PMC3447870; doi:10.1371/journal.pone.0045410)
Supplement: Table S1 — Expression values and fold changes of the most abundant miRNAs in undifferentiated and differentiated VSC. (DOC) [file pone.0045410.s005.doc]

**Table S1. Expression values and fold changes of the most abundant miRNAs in undifferentiated and differentiated VSC.**

|  | sequencing expression values | | | | normalized expression values | | | | fold changes | |
| --- | --- | --- | --- | --- | --- | --- | --- | --- | --- | --- |
|  | IVSC | IVSC-MDI | SVSC | SVSC-MDI | IVSC | IVSC-MDI | SVSC | SVSC-MDI | IVSC-MDI/IVSC | SVSC-MDI/SVSC |
| let-7a | 212934 | 221014 | 814656 | 53235 | 9862 | 10657 | 31667 | 10304 | 1.08 | -3.07 |
| let-7c | 22249 | 26019 | 48766 | 10602 | 1030 | 1255 | 1896 | 2052 | 1.22 | 1.08 |
| let-7e | 12204 | 22122 | 26287 | 9809 | 565 | 1067 | 1022 | 1899 | 1.89 | 1.86 |
| let-7f | 401351 | 521213 | 851683 | 97825 | 18589 | 25132 | 33106 | 18935 | 1.35 | -1.75 |
| let-7g | 51937 | 83534 | 106379 | 11110 | 2406 | 4028 | 4135 | 2150 | 1.67 | -1.92 |
| mir-100 | 30054 | 31863 | 134779 | 4589 | 1392 | 1536 | 5239 | 888 | 1.10 | -5.90 |
| mir-101 | 107978 | 89106 | 78989 | 14694 | 5001 | 4296 | 3070 | 2844 | -1.16 | -1.08 |
| mir-103 | 70805 | 80642 | 248601 | 11464 | 3279 | 3888 | 9664 | 2219 | 1.19 | -4.36 |
| mir-10b | 52330 | 103683 | 27439 | 39753 | 2424 | 4999 | 1067 | 7694 | 2.06 | 7.21 |
| mir-125b | 23966 | 48940 | 31107 | 13027 | 1110 | 2360 | 1209 | 2521 | 2.13 | 2.09 |
| mir-145 | 12630 | 77415 | 13878 | 17916 | 585 | 3733 | 539 | 3468 | 6.38 | 6.43 |
| mir-148a | 585406 | 447302 | 292284 | 110252 | 27114 | 21568 | 11362 | 21340 | -1.25 | 1.88 |
| mir-152 | 94764 | 45153 | 117295 | 6986 | 4389 | 2177 | 4559 | 1352 | -2.02 | -3.37 |
| mir-206 | 64111 | 2057 | 50 | 4 | 2969 | 99 | 2 | 1 | -29.94 | -2.51 |
| mir-21 | 3902324 | 4949407 | 7860307 | 672825 | 180742 | 238647 | 305543 | 130229 | 1.32 | -2.34 |
| mir-23a | 94649 | 126741 | 74150 | 26345 | 4384 | 6111 | 2882 | 5099 | 1.39 | 1.77 |
| mir-24 | 55212 | 71641 | 84731 | 9791 | 2557 | 3454 | 3294 | 1895 | 1.35 | -1.74 |
| mir-27a | 76739 | 87354 | 110232 | 27020 | 3554 | 4212 | 4285 | 5230 | 1.19 | 1.22 |
| mir-30a | 36558 | 97468 | 14925 | 8945 | 1693 | 4700 | 580 | 1731 | 2.78 | 2.98 |
| mir-30d | 59098 | 92339 | 162408 | 8710 | 2737 | 4452 | 6313 | 1686 | 1.63 | -3.74 |
| mir-30e | 62466 | 84170 | 100215 | 4140 | 2893 | 4058 | 3896 | 801 | 1.40 | -4.86 |
| mir-378 | 3453011 | 1898717 | 2338977 | 454315 | 159931 | 91551 | 90920 | 87935 | -1.75 | -1.03 |
|  |  |  |  |  |  |  |  |  |  |  |
