# Supplementary material for: MicroRNAome Comparison between Intramuscular and Subcutaneous Vascular Stem Cell Adipogenesis
Source: PLoS One. 2012 Sep 20;7(9):e45410. doi: 10.1371/journal.pone.0045410 (PMC3447870; doi:10.1371/journal.pone.0045410)
Supplement: Table S3 — QPCR primers of mRNAs. (DOC) [file pone.0045410.s007.doc]

**Table S3. QPCR primers of mRNAs.**

|  | Fordward | Reverse |
| --- | --- | --- |
| GAPDH | ACAGTCAAGGCGGAGAACG | GGCAGAAGGGGCAGAGAT |
| 18sRNA | GCTGCCTTCCTTGGATGTG | TTCGATGGTAGTCGCCGT |
| Wnt10b | CTGTCCCGAGGCAAGAGTTT | GCATTTCCGCTTCAGATTTT |
| C/EBPβ | GGTGGACAAGCACAGCGA | TGCTGCGTCTCCAGGTTG |
| PPARγ1 | CCATTCGCATCTTTCAGGG | CGTGGACGCCATACTTTAGGA |
| PPARγ2 | TTGATTTCTCCAGCATTTCC | GGCTCCACTTTGATGGCACT |
| C/EBPα | TGGACAAGAACAGCAACGAG | ACCTTCTGTTGAGTCTCCACG |
| GPDH | GGGGCTGGCTTCTGTGAT | AATGGACTTTCCTGTGCGG |
| ap2/FABP4 | GGAAACTTGTCTCCAGTGAAAAC | TGGTGCTCTTGACTTTCCTGT |
| ACCA | GGTTATGTGAAGGATGTGGATGA | CCTGTCTGAAGAGGTTAGGGAAG |
| ACCB | GTGGAGGGAAAGGAATACGG | GAACAGTCACGCCCAAACA |
| FASN | GAAGGACCACAGGGACAACC | ATAGACGGCGACAGAGGAGC |
| SCD | ATGACATCTATGACCCAACCTACC | GCAAACGCCCAGAGCAAG |
| HSL | CTCCTCGTGGCTCAACTCC | GCCGCATTGGCTCTTCTG |
| SREBP1 | ACCCCACCAGTCCTGATGC | TACATCTTCAGCGGGGTGG |
| FABP5 | GAAGGAAGTAGGAGTGGGAATG | TTTGATGGTGAGGTCTTTGCC |
| LPL | CAAACTTGTGGCTGCCCTAT | AGGTGGACATTGTTCGGAGG |
| Leptin | GGAGAGTCCAGGATGACACCA | CAGGGATGAAGTCCAAACCG |
