# Supplementary material for: MicroRNAome Comparison between Intramuscular and Subcutaneous Vascular Stem Cell Adipogenesis
Source: PLoS One. 2012 Sep 20;7(9):e45410. doi: 10.1371/journal.pone.0045410 (PMC3447870; doi:10.1371/journal.pone.0045410)
Supplement: Table S4 — QPCR primers of miRNAs. (DOC) [file pone.0045410.s008.doc]

**Table S4. QPCR primers of miRNAs.**

| Universal primer | TGCTGTCAACGATACGCTACG |
| --- | --- |
| ssc-miR-27a-F | TTCACAGTGGCTAAGTTCCGCA |
| ssc-miR-27b-F | TTCACAGTGGCTAAGTTCTGCA |
| ssc-miR-210-F | CTGTGCGTGTGACAGCGGCTGAA |
| ssc-miR-100-F | AACCCGTAGATCCGAACTTGTGA |
| ssc-miR-103-F | AGCAGCATTGTACAGGGCTATGAA |
| ssc-miR-125a-F | TCCCTGAGACCCTTTAACCTGTGA |
| ssc-miR-140-F | AGTGGTTTTACCCTATGGTAGA |
| ssc-miR-145-F | GTCCAGTTTTCCCAGGAATCCCTTA |
| ssc-miR-152-F | TCAGTGCATGACAGAACTTGGA |
| ssc-miR-26a-F | TTCAAGTAATCCAGGATAGGCTA |
| ssc-miR-30c-F | TGTAAACATCCTACACTCTCAGCA |
| ssc-miR-30d-F | TGTAAACATCCCCGACTGGAAGCTA |
| ssc-miR-378-F | ACTGGACTTGGAGTCAGAAGGCA |
| ssc-miR-99a-F | AACCCGTAGATCCGATCTTGTGA |
| ssc-miR-143-3p-F | TGAGATGAAGCACTGTAGCTCA |
| ssc-miR-19b-F | TGTGCAAATCCATGCAAAACTGAA |
| ssc-miR-107-F | AGCAGCATTGTACAGGGCTATCAA |
| ssc-miR-21-F | TAGCTTATCAGACTGATGTTGAA |
