# Supplementary material for: MicroRNAome Comparison between Intramuscular and Subcutaneous Vascular Stem Cell Adipogenesis
Source: PLoS One. 2012 Sep 20;7(9):e45410. doi: 10.1371/journal.pone.0045410 (PMC3447870; doi:10.1371/journal.pone.0045410)
Supplement: Text S1 — The procedures for isolation, plating and differentiation of VSC cells. (DOC) [file pone.0045410.s009.doc]

**Text S1. The procedures for isolation, plating and differentiation of VSC cells.** Cells from the primary stromal vascular fraction (SVF) were isolated according to the method established previously with modifications. Immediately after the dorsal longissimus dorsi and subcutaneous fat tissue were dissected, they were extensively washed 3 times with phosphate-buffered saline (PBS) containing 1000 IU/ml penicillin-streptomycin (Sigma Aldrich), and visible blood vessels and connective tissues were removed. The washed tissues were minced into small pieces in Dulbecco’s modified Eagle’s medium/Nutrient Mixture F-12 Ham (DMEM/F12, pH 7.4, Sigma Aldrich) containing 0.1% (w/v) type I collagenase (Sigma Aldrich) for subcutaneous fat tissue or 0.2% type II collagenase (Sigma Aldrich) for dorsal longissimus dorsi, and 2% bovine serum albumin (Fraction V, Sigma Aldrich) supplemented with 100 IU/ml penicillin-streptomycin. The finely cut tissues were digested for 1h at 37^o^C by shaking gently in a water bath, and the disrupted tissue was filtered through 250 μm and 150 μm nylon meshes to remove undigested tissue, and the filtered cells were washed 3 times with DMEM/F12 by centrifugation at 500 g for 5 minutes. The SVF cells in the pellet at the bottom of the tubes were resuspended in DMEM/F12 containing 20% fetal bovine serum (FBS, PAA Laboraties GmbH, Pasching, Austria) and 100 IU/ml penicillin-streptomycin. The subcutaneous SVF cells were seeded on to 6-well plates at a density of 10^6^ cells/well, and cultured in a 5% CO_2_ incubator at 37^o^C for 1h to allow preadipocytes to attach to the plates . The cells were washed 3 times with PBS and the non-adherent cells removed, fresh growth medium was added and the adherent vascular stem cells (VSC) were cultured in a 5% CO_2_ incubator at 37^o^C for 10 h. Cells that did not adhere well to the plate were removed, and the VSC were incubated under the above conditions until confluent. To maintain similar times required for cells to reach confluent densities, intramuscular SVF cells were plated at a density of 1.5×10^6^ cells/well. Two days after the cells reached confluence, differentiation of the cells was induced by changing the DMEM/F12 medium into DMEM/F12 differentiation medium containing 20% FBS, 0.5 mM 3-isobutyl-1-methylxanthine (IBMX, Sigma Aldrich), 0.25 μM dexamethasone (Sigma Aldrich), 5 mg/l insulin (Sigma Aldrich) and 100 IU/ml penicillin-streptomycin . The differentiation medium was replaced by DMEM/F12 medium without IBMX and dexamethasone after three days’ culture. At day 6, the medium was changed to DMEM/F12 growth medium supplemented with 20% FBS and 100 IU/ml penicillin-streptomycin, and the cells were allowed to differentiate for a further 3 or 4 days.

1. Hausman GJ, Poulos S (2004) Recruitment and differentiation of intramuscular preadipocytes in stromal-vascular cell cultures derived from neonatal pig semitendinosus muscles. Journal of Animal Science 82: 429-437.

2. Hausman GJ, Poulos SP (2005) A method to establish co-cultures of myotubes and preadipocytes from collagenase digested neonatal pig semitendinosus muscles. Journal of Animal Science 83: 1010-1016.

3. Zhou X, Li D, Yin J, Ni J, Dong B, et al. (2007) CLA differently regulates adipogenesis in stromal vascular cells from porcine subcutaneous adipose and skeletal muscle. J Lipid Res 48: 1701-1709.

4. Yu ZK, Hausman GJ (1998) Preadipocyte screening by laminin in porcine stromal vascular cell cultures. Obes Res 6: 299-306.

5. Kano K, Nobusue H (2010) Establishment and Characteristics of Porcine Preadipocyte Cell Lines Derived From Mature Adipocytes. Journal of Cellular Biochemistry 109: 542-552.
